# Supplementary material for: Glioma-neuronal circuit remodeling induces regional immunosuppression
Source: Nat Commun. 2025 May 22;16:4770. doi: 10.1038/s41467-025-60074-z (PMC12098748; doi:10.1038/s41467-025-60074-z)
Supplement: Supplementary file 1 — Supplementary Information [file 41467_2025_60074_MOESM1_ESM.pdf]

Glioma-neuronal circuit remodeling induces regional immunosuppression

Takahide Nejo, Saritha Krishna, Akane Yamamichi, Senthilnath Lakshmanachetty, Christian Jimenez, Kevin Y. Lee, Donovan L. Baker, Jacob S. Young, Tiffany Chen, Su Su Sabai Phyu, Lan Phung, Marco Gallus, Gabriella C. Maldonado, Kaori Okada, Hirokazu Ogino, Payal B. Watchmaker, David Diebold, Abrar Choudhury, Andy G. S. Daniel, Cathryn R. Cadwell, David R. Raleigh, Shawn L. Hervey-Jumper, Hideho Okada

Supplementary Information

Supplementary Figures and Legends

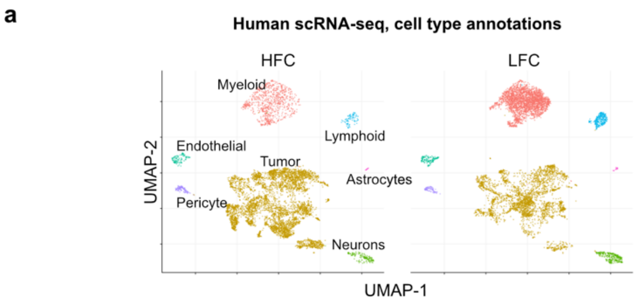

	patient 1			patient 2			patient 3			whole dataset		
annotation	HFC	LFC	total	HFC	LFC	total	HFC	LFC	total	HFC	LFC	total
Tumor Cells	525	1250	1775	2143	406	2549	2657	1556	4213	5325	3212	8537
Myeloid Cells	309	2338	2647	294	81	375	248	505	753	851	2924	3775
Neurons	37	276	313	54	49	103	60	2	62	151	327	478
Lymphoid Cells	64	284	348	10	7	17	12	18	30	86	309	395
Endothelial Cells	12	67	79	5	1	6	100	95	195	117	163	280
Pericytes	19	33	52	15	1	16	82	55	137	116	89	205
Astrocytes	1	29	30	8	4	12	11	8	19	20	41	61

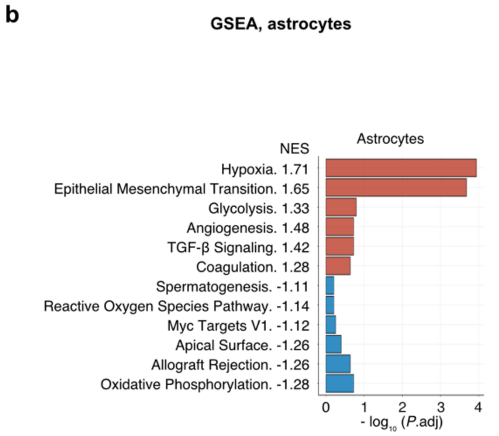

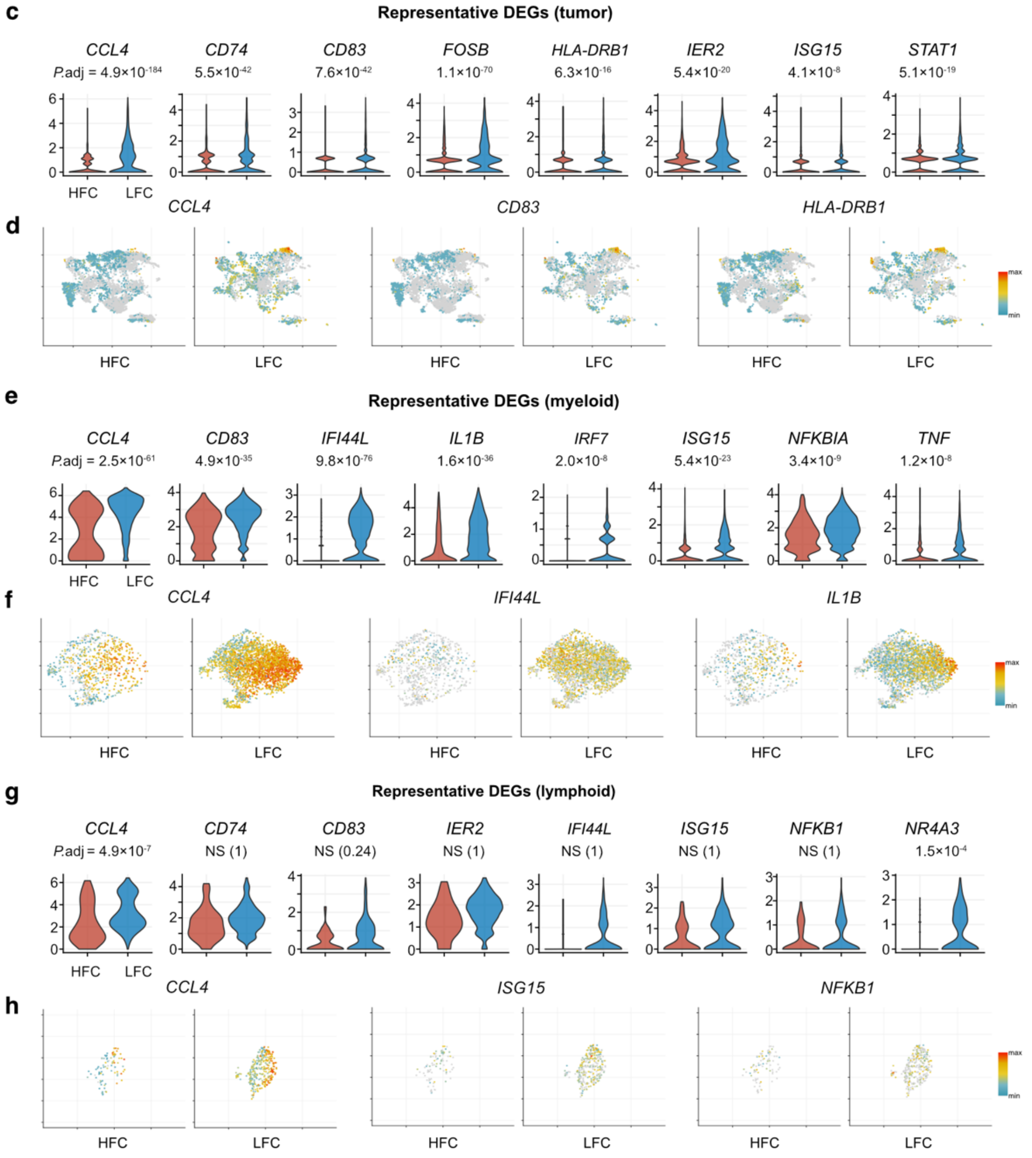

Supplementary Fig. 1 | Single-cell RNA-seq analysis of clinical samples annotated as HFC- or LFC-derived (Related to Fig. 1)

a, UMAP plots showing each identified cell cluster, as previously reported[1]. The corresponding table shows the breakdown of the number of cells within the dataset.

b, Bar plot summarizing the result of GSEA with MSigDB Hallmark collection comparing HFC vs. LFC within astrocytes. Statistical values are shown in each figure as normalized enrichment scores (NES) and adjusted *P* values. Positive and negative NES values indicate upregulation (shown in red) and downregulation (shown in blue) in HFC compared with those from LFC regions. The top six upregulated and downregulated signatures are presented. The complete results of the GSEA are provided in **Supplementary Data 1**.
c–h, Violin plots (**c, e, g**) and feature plots (**d, f, h**) showing representative leading-edge genes differentially expressed in tumor (**c–d**), myeloid (**e–f**), and lymphoid (**g–h**) cells between HFC and LFC regions. Values indicate adjusted *P* values.
P values were calculated using the MAST algorithm with Benjamini-Hochberg adjustment (**c, e, g**). NS, not significant.

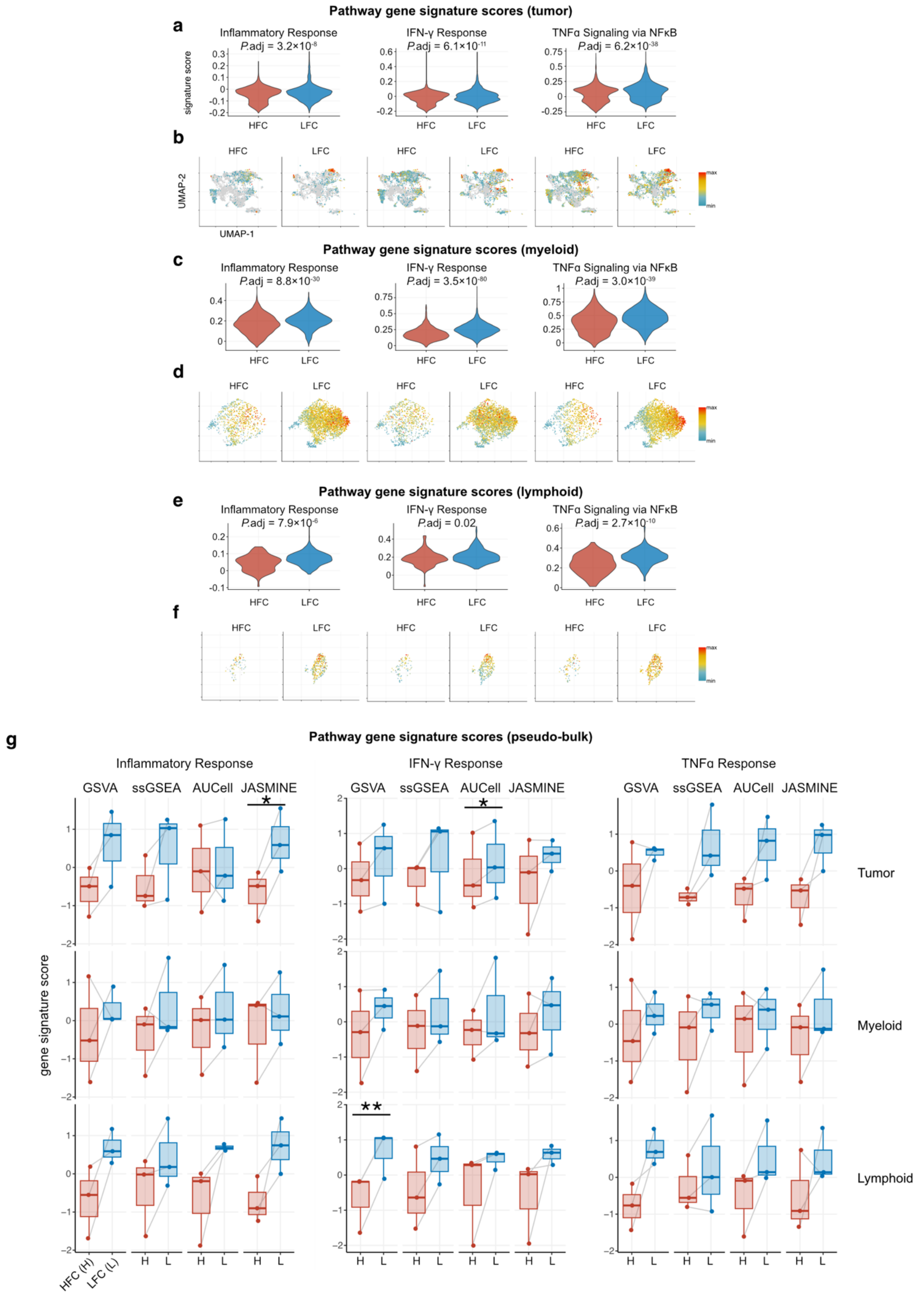

Supplementary Fig. 2 | Gene signature score analysis across cell populations (Related to Fig. 1)
a–f, Violin plots (**a, c, e**), and feature plots (**b, d, f**) showing the signature scores of *Inflammatory Response*, *Interferon-γ Response*, and *TNFα signaling via NFκB pathways* (all from MSigDB

Hallmark collection) (tumor: n = 5,325 HFC and 3,212 LFC cells; myeloid: n = 851 HFC and 2,924 LFC cells; lymphoid: n = 86 HFC and 309 LFC cells). In feature plots, signature score expression values are indicated in the color bar.

g, Box plots showing the distribution of the same signature scores calculated for pseudo-bulk gene expression matrices (3 HFC vs. 3 LFC) in each cell population. Signature scores were calculated using the following four algorithms: GSVA, ssGSEA[2], AUCell[3], and JASMINE[4]. Gray lines connect paired HFC and LFC samples.

P values were calculated using the Wilcoxon rank-sum test for single-cell comparisons with Benjamini-Hochberg correction (**a, c, e**), and Welch's unpaired *t*-test for pseudo-bulk comparisons (**g**). $^{*}P < 0.05$, $^{**}P < 0.01$.

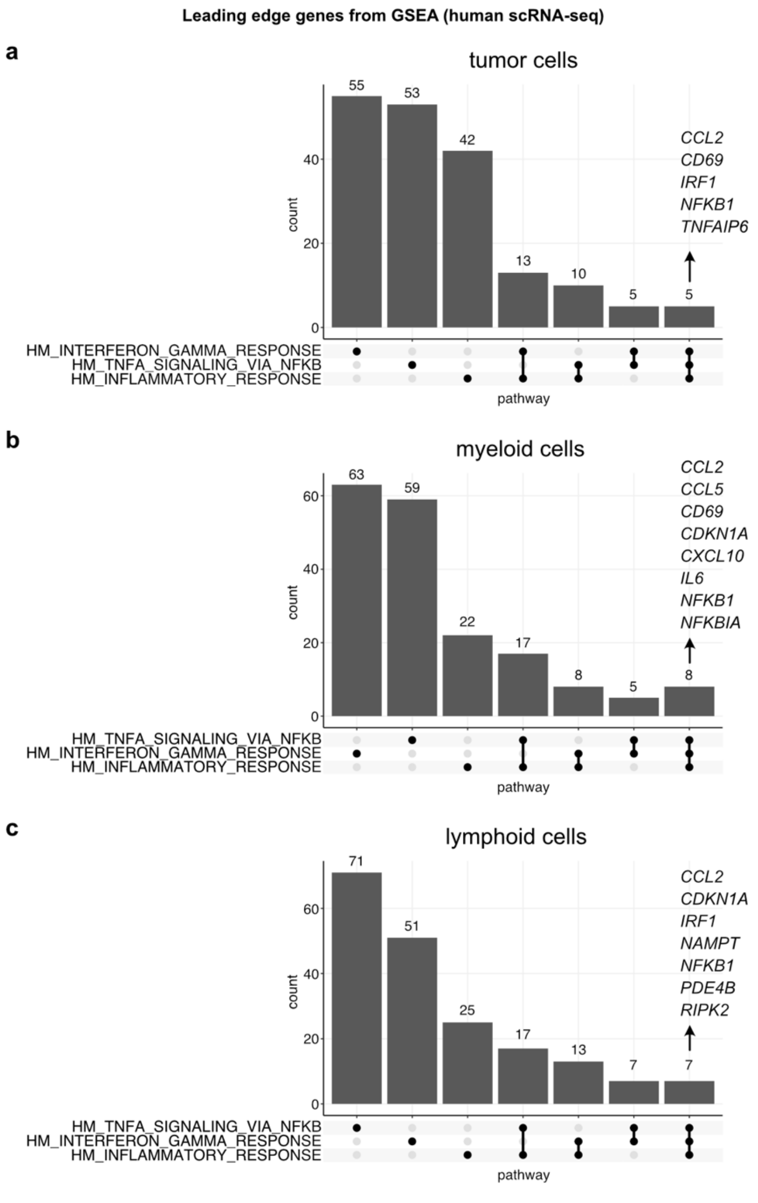

Supplementary Fig. 3 | Shared leading-edge genes identified through GSEA (Related to Fig. 1)

a-c, Upset plots showing the number of leading-edge genes that are either shared across multiple pathways or unique to a specific pathway, identified through GSEA for tumor cells (**a**), myeloid cells (**b**), and lymphoid cells (**c**). The genes identified in all three pathways—*Inflammatory Response*,

Interferon-γ Response, *TNFa signaling via NFκB pathways* (all from MSigDB Hallmark collection)––are annotated. Source data are provided as a Source Data file.

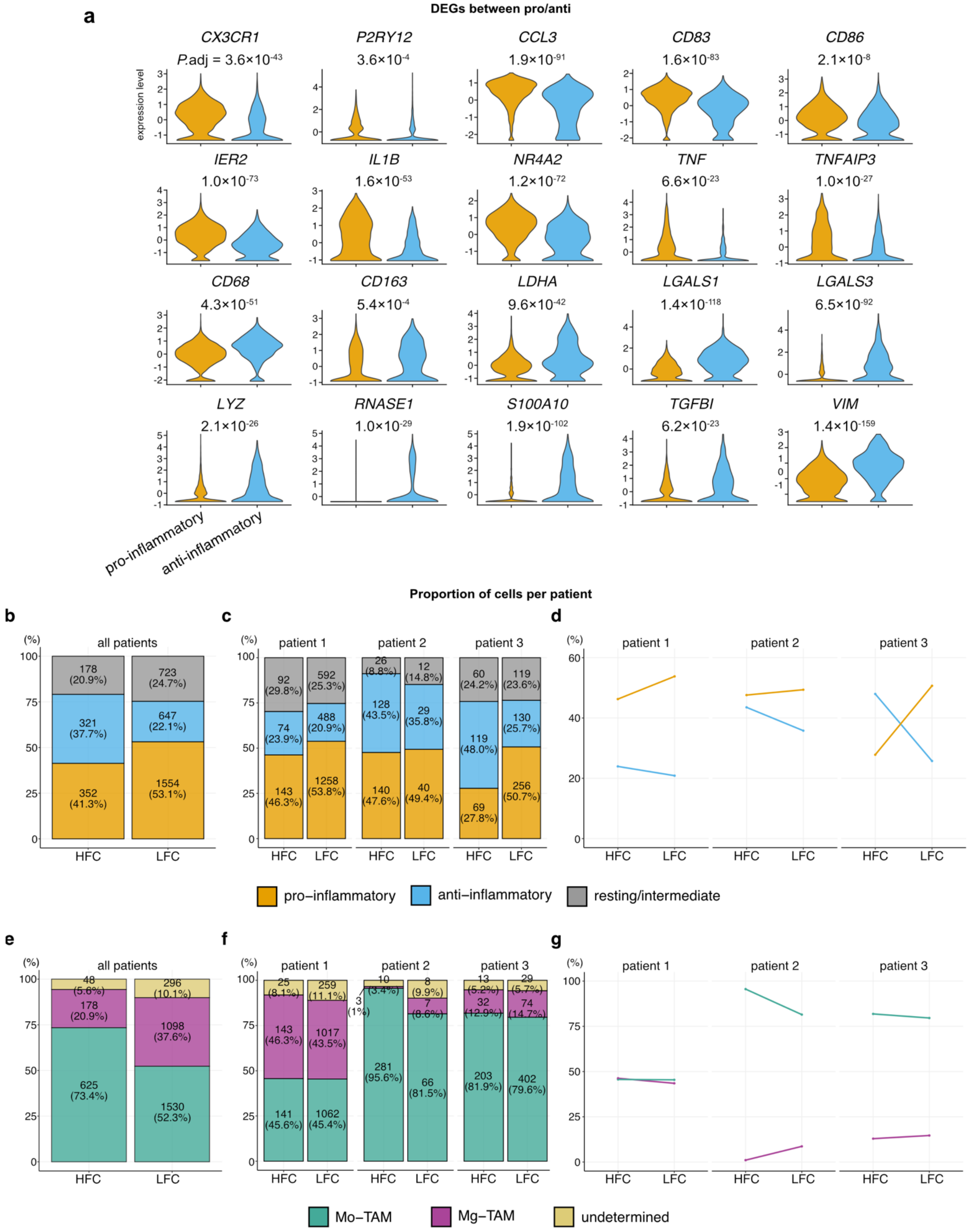

Supplementary Fig. 4 | Detailed characterization of TAMs (Related to Fig. 1)
a, Violin plots showing the distribution and relative expression levels of representative pro- and anti-inflammatory marker genes differentially expressed between the estimated pro- and anti-inflammatory TAM groups.
b–d, Bar plots and line plots displaying the percentages of pro-inflammatory, anti-inflammatory, and undetermined subpopulations identified within the entire myeloid cell population (**b**), and within HFC- and LFC-derived cells from each patient (**c**). The line plot summarizes these percentages within each patient's sample (**d**).
e–g, Bar plots and line plots displaying the percentages of microglia-derived (Mg-)TAMs, monocyte-derived (Mo-)TAMs, and undetermined subpopulations identified within the entire myeloid cell population (**e**), and within HFC- and LFC-derived cells from each patient (**f**). The line plot summarizes these percentages within each patient's sample (**g**).
P values were calculated using the MAST algorithm with Benjamini-Hochberg correction.

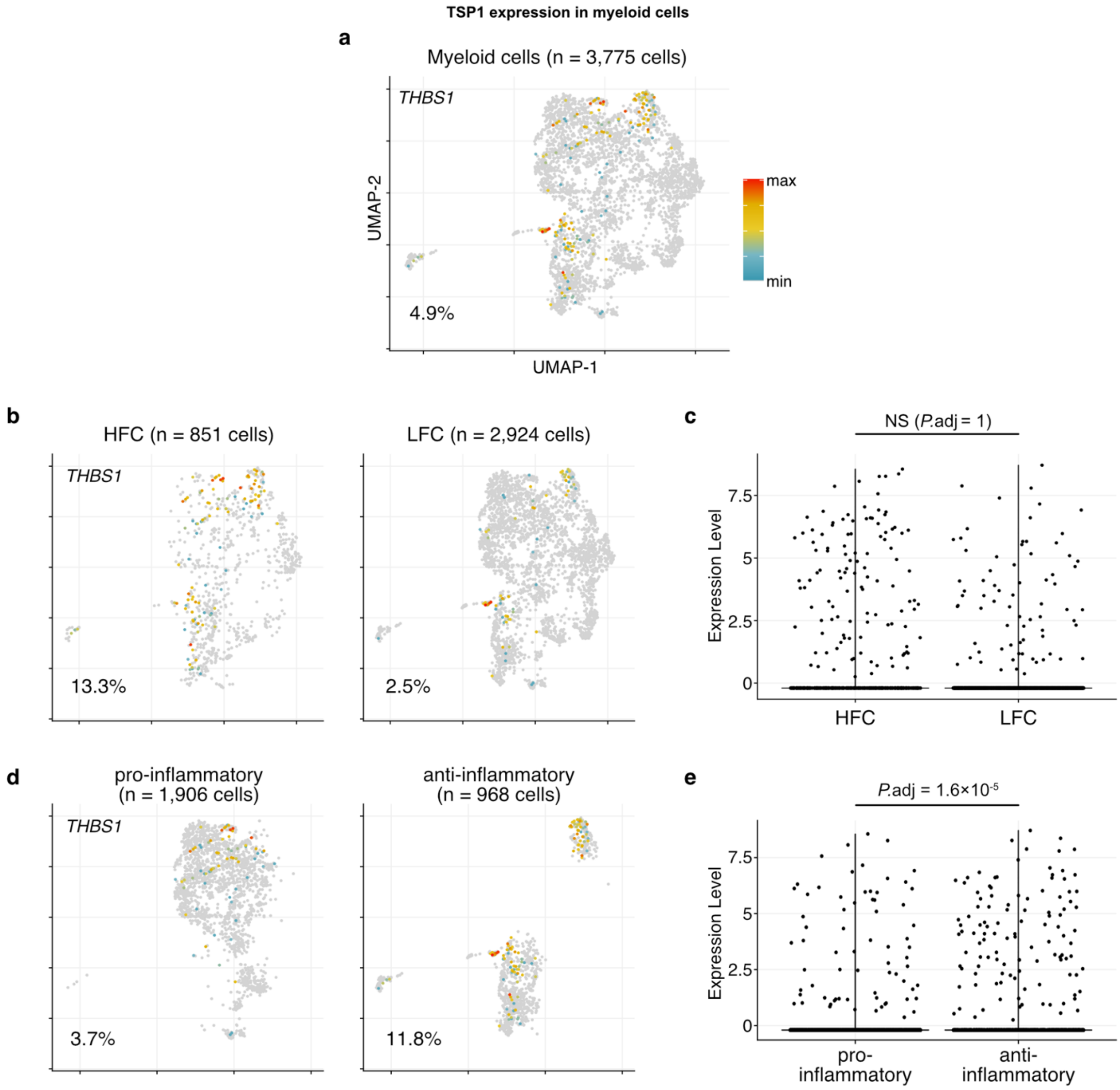

Supplementary Fig. 5 | TSP1 gene expression status in TAMs (Related to Fig. 1)
Feature plots and violin plots displaying TSP1 (*THBS1*) gene expression in whole myeloid cells (n = 3,775 cells) (**a**), the HFC and LFC regions (n = 851 and 2,924 cells, respectively) (**b, c**), and pro- and anti-inflammatory populations (n = 1,906 and 968 cells, respectively) (**d, e**).
P values were calculated using the MAST algorithm with Benjamini-Hochberg correction (**a**).
NS, not significant.

Visium spatial transcriptome of human glioblastoma

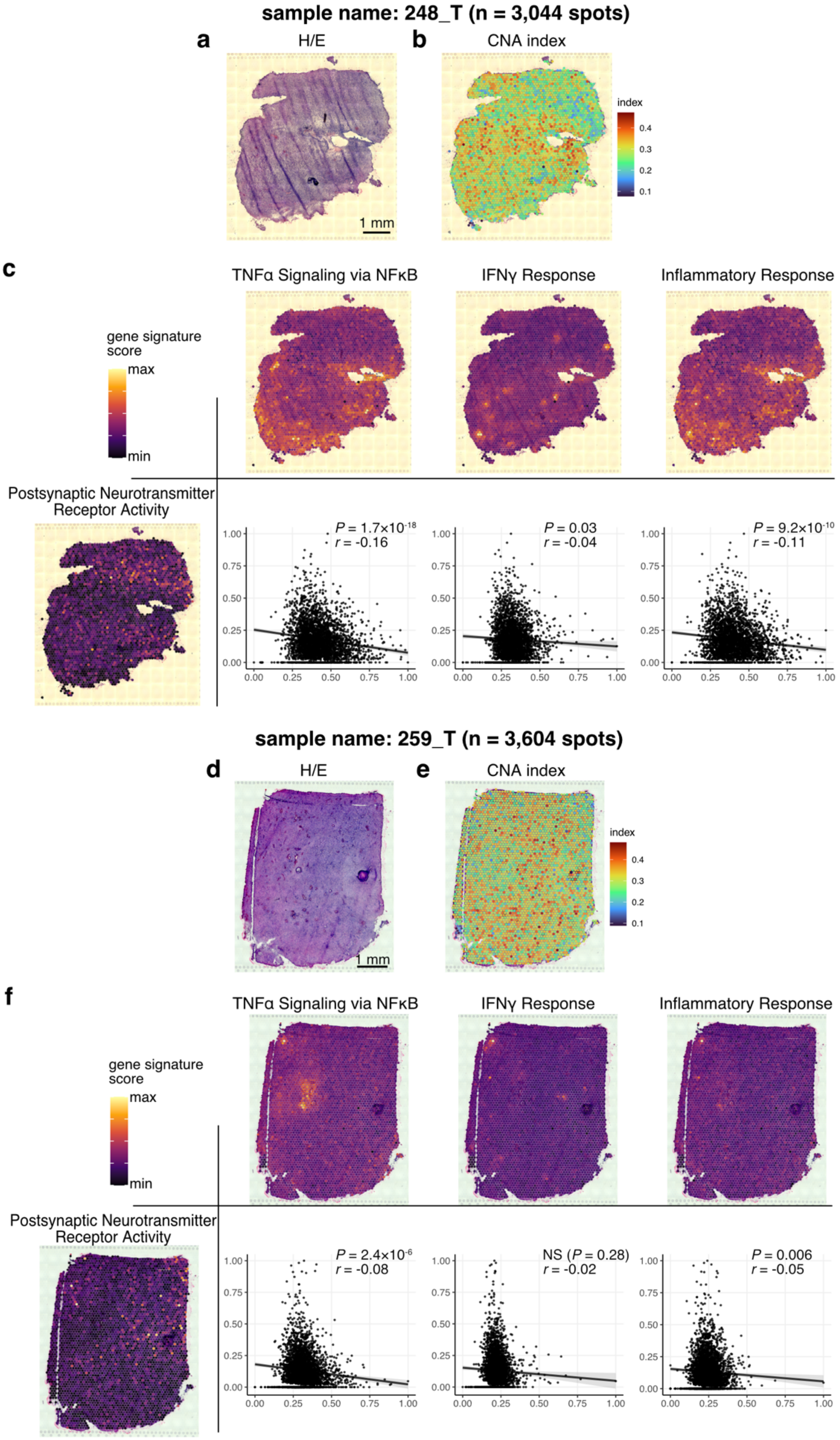

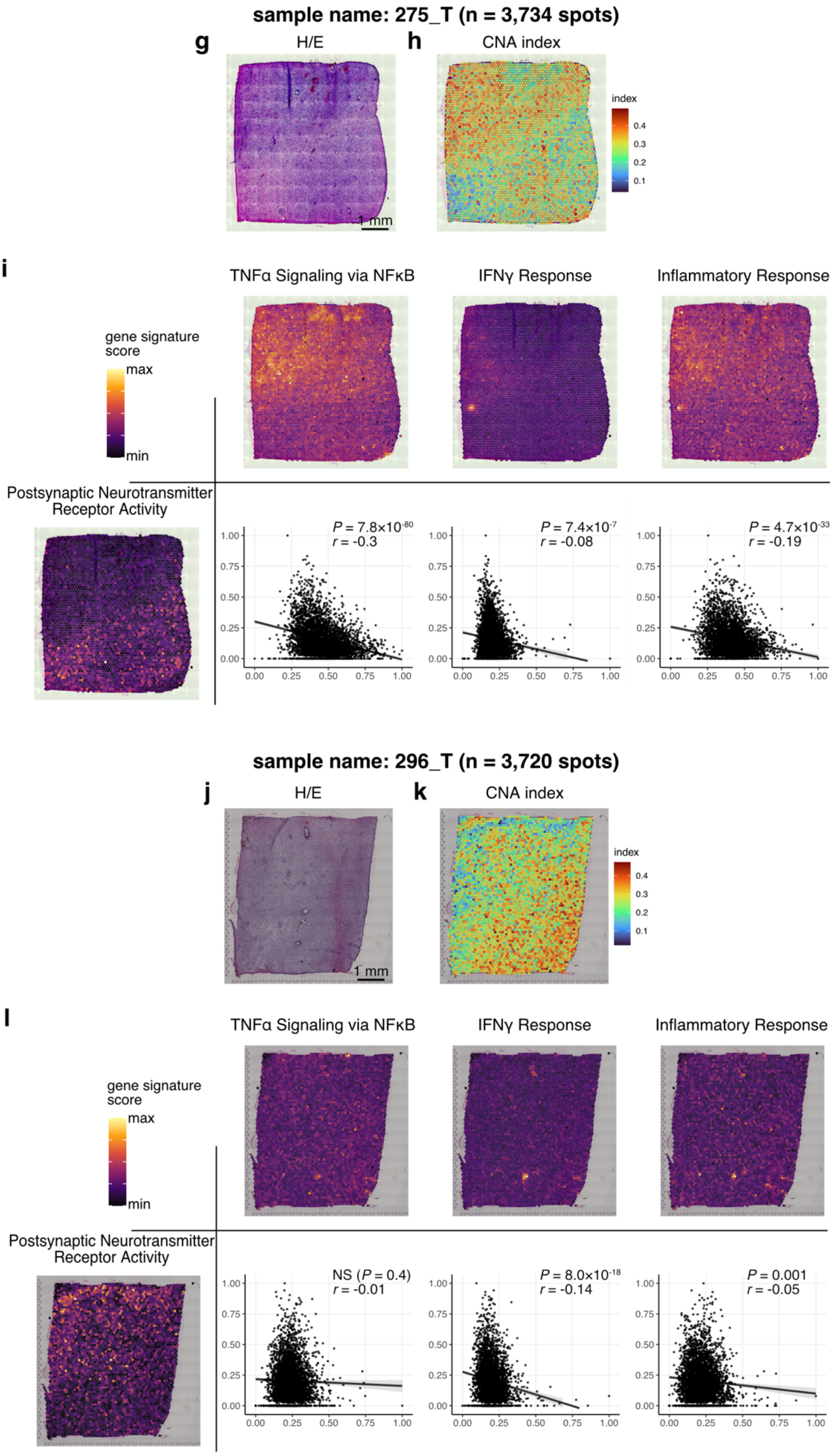

sample name: 275_T (n = 3,734 spots)
g
H/E
1 mm
h
CNA index
index
0.4
0.3
0.2
0.1
i
TNFα Signaling via NFκB
IFNγ Response
Inflammatory Response
gene signature score
max
min
Postsynaptic Neurotransmitter Receptor Activity
P = 7.8×10-80
r = -0.3
P = 7.4×10-7
r = -0.08
P = 4.7×10-33
r = -0.19
0.00 0.25 0.50 0.75 1.00
sample name: 296_T (n = 3,720 spots)
j
H/E
1 mm
k
CNA index
index
0.4
0.3
0.2
0.1
l
TNFα Signaling via NFκB
IFNγ Response
Inflammatory Response
gene signature score
max
min
Postsynaptic Neurotransmitter Receptor Activity
NS (P = 0.4)
r = -0.01
P = 8.0×10-18
r = -0.14
P = 0.001
r = -0.05
0.00 0.25 0.50 0.75 1.00

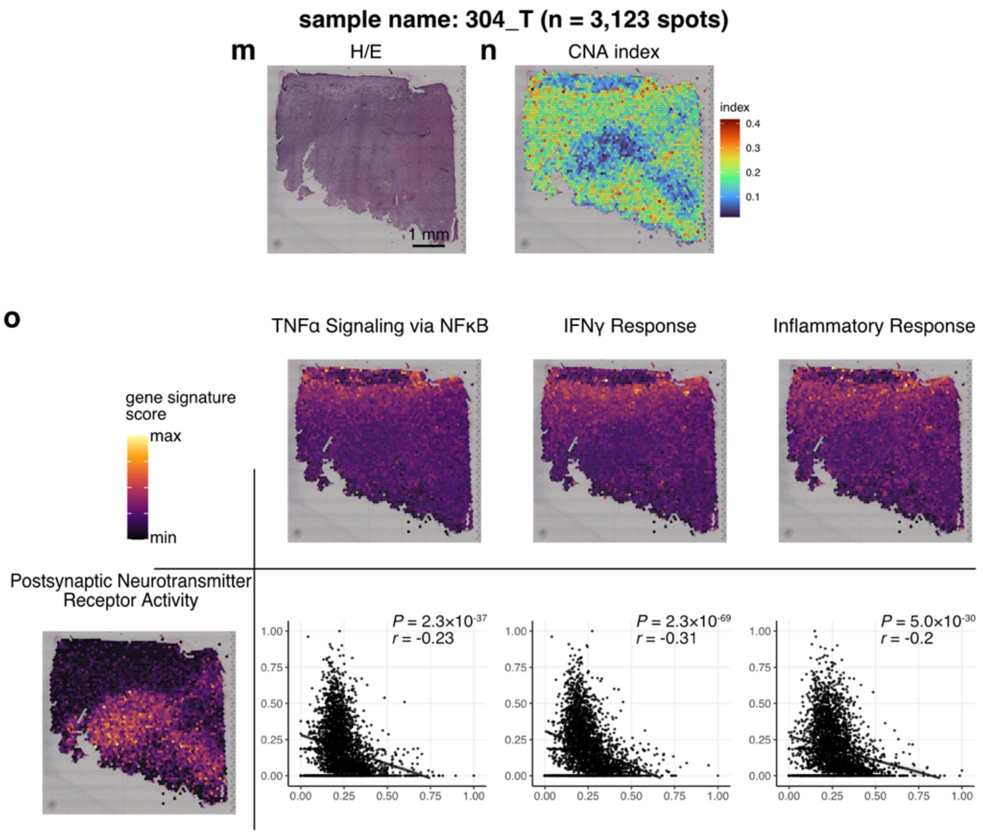

Supplementary Fig. 6 | Spatial transcriptomic analyses of human glioblastoma. (Related to Fig. 2)

Summary of spatial transcriptomic analysis results for five additional human glioblastoma specimens: 248_T (**a**–**c**), 259_T (**d**–**f**), 275_T (**g**–**i**), 296_T (**j**–**l**), and 304_T (**m**–**o**), obtained from SPATAData[5,6].

a, d, g, j, m, H&E-stained histological images.

b, e, h, k, n, Surface plots showing the distribution of CNA index in each specimen.

c, f, i, l, o, Surface plots displaying gene set enrichment signature scores for *Postsynaptic Neurotransmitter Receptor Activity* (GO:MF), *TNFa-Signaling via NFκB*, *IFNγ Response*, and *Inflammatory Response* (all from Hallmark) within each specimen. Scatter plots show the correlations between *Post-synaptic Neurotransmitter Receptor Activity* and the other pathways. *P* values are calculated using Spearman's correlation test. *r*, correlation coefficient. NS, not significant. Source data are provided as a Source Data file.

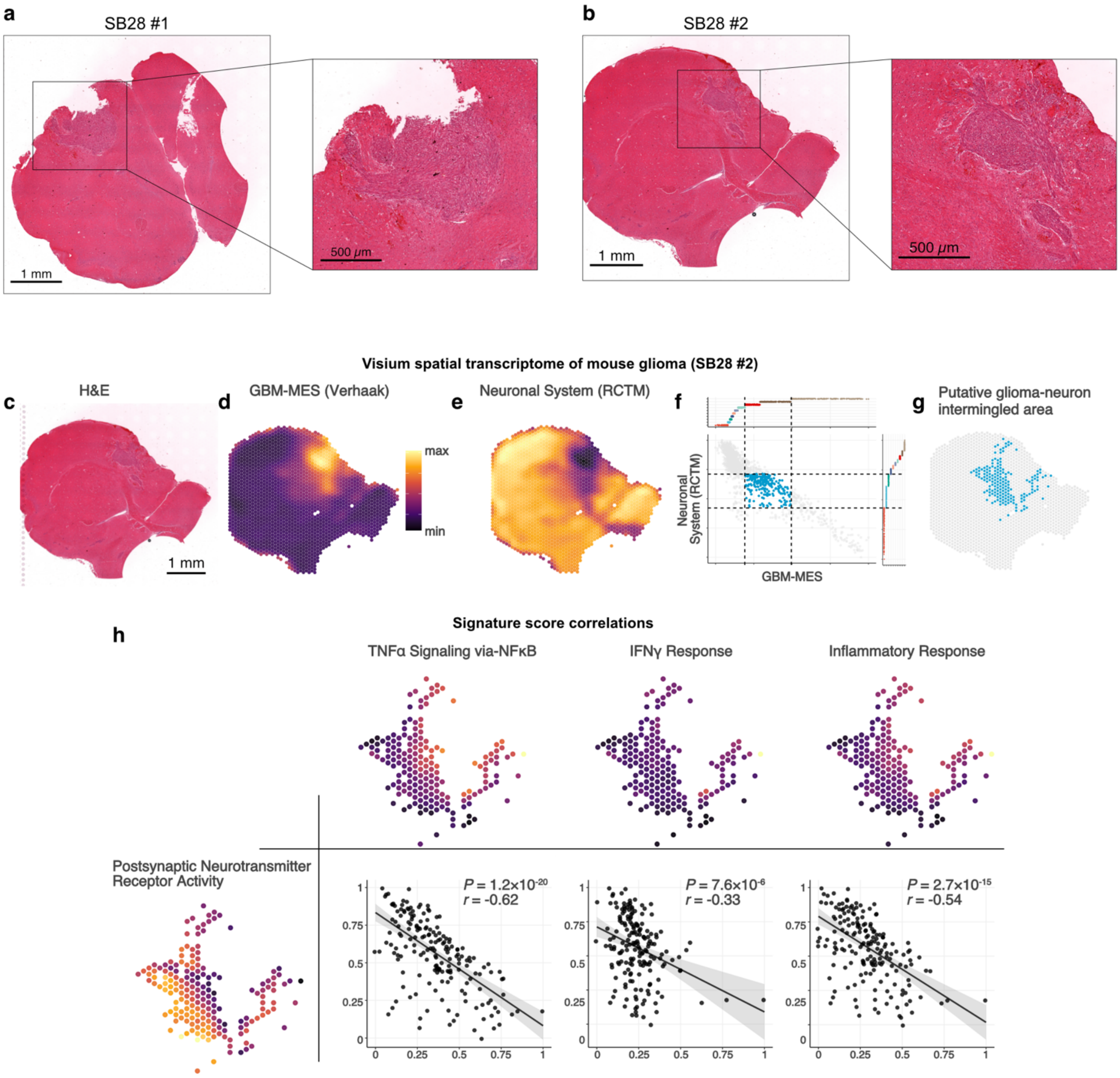
a
SB28 #1
1 mm
500 μm
b
SB28 #2
1 mm
500 μm
Visium spatial transcriptome of mouse glioma (SB28 #2)
c
H&E
1 mm
d
GBM-MES (Verhaak)
max
min
e
Neuronal System (RCTM)
f
Neuronal System (RCTM)
GBM-MES
g
Putative glioma-neuron intermingled area
h
Signature score correlations
TNFα Signaling via-NFκB
IFNγ Response
Inflammatory Response
Postsynaptic Neurotransmitter Receptor Activity
P = 1.2x10^-20
r = -0.62
P = 7.6x10^-6
r = -0.33
P = 2.7x10^-15
r = -0.54

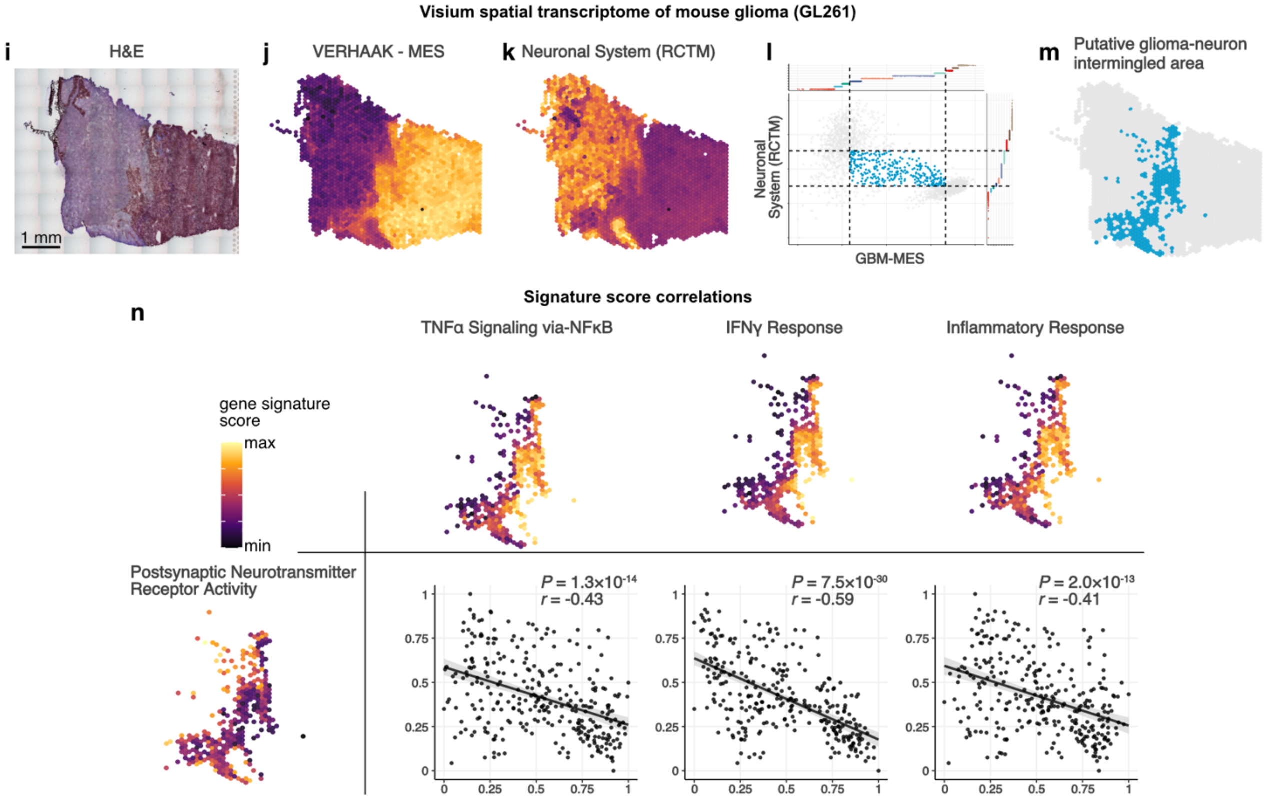

Supplementary Fig. 7 | Spatial transcriptomic analyses of mouse glioblastoma models. (Related to Fig. 2)

a–b, High-resolution H&E staining images of two SB28-tumor-bearing mouse brains.

c–n, Summary of spatial transcriptomic analyses of two additional mouse glioma models: a second SB28 tumor-bearing mouse brain (**c–h**), and a GL261 tumor-bearing mouse brain (**i–n**) obtained from GSE245263[7].

c, i, H&E-stained histological images.

d–e, j–k, Surface plots showing gene set enrichment scores of *Verhaak Glioblastoma Mesenchymal ('GBM-MES')* (C2:CGP) (**d, j**) and *Neuronal System* (Reactome) (**e, k**).

f–g, l–m, Scatter plots displaying the correlation between *GBM-MES* and *Neuronal System* scores across the dataset, highlighting spots with upper 10–30 percentiles of *GBM-MES* scores and lower 10–30 percentiles of *Neuronal System* (blue) (**f, l**). Surface plots show the 'putative glioma-neuronal infiltration areas' within the tumor bed based on these distributions (**g, m**).

h, n, Surface plots showing enrichment scores for *Postsynaptic Neurotransmitter Receptor Activity* (GO:MF), *TNFa-Signaling via NFκB*, *IFNγ Response*, and *Inflammatory Response* (all from Hallmark) within each specimen. Scatter plots display correlations between *Postsynaptic Neurotransmitter Receptor Activity* and the other pathways.

P values were calculated using Pearson's correlation test. *r*, correlation coefficient.

Source data are provided as a Source Data file.

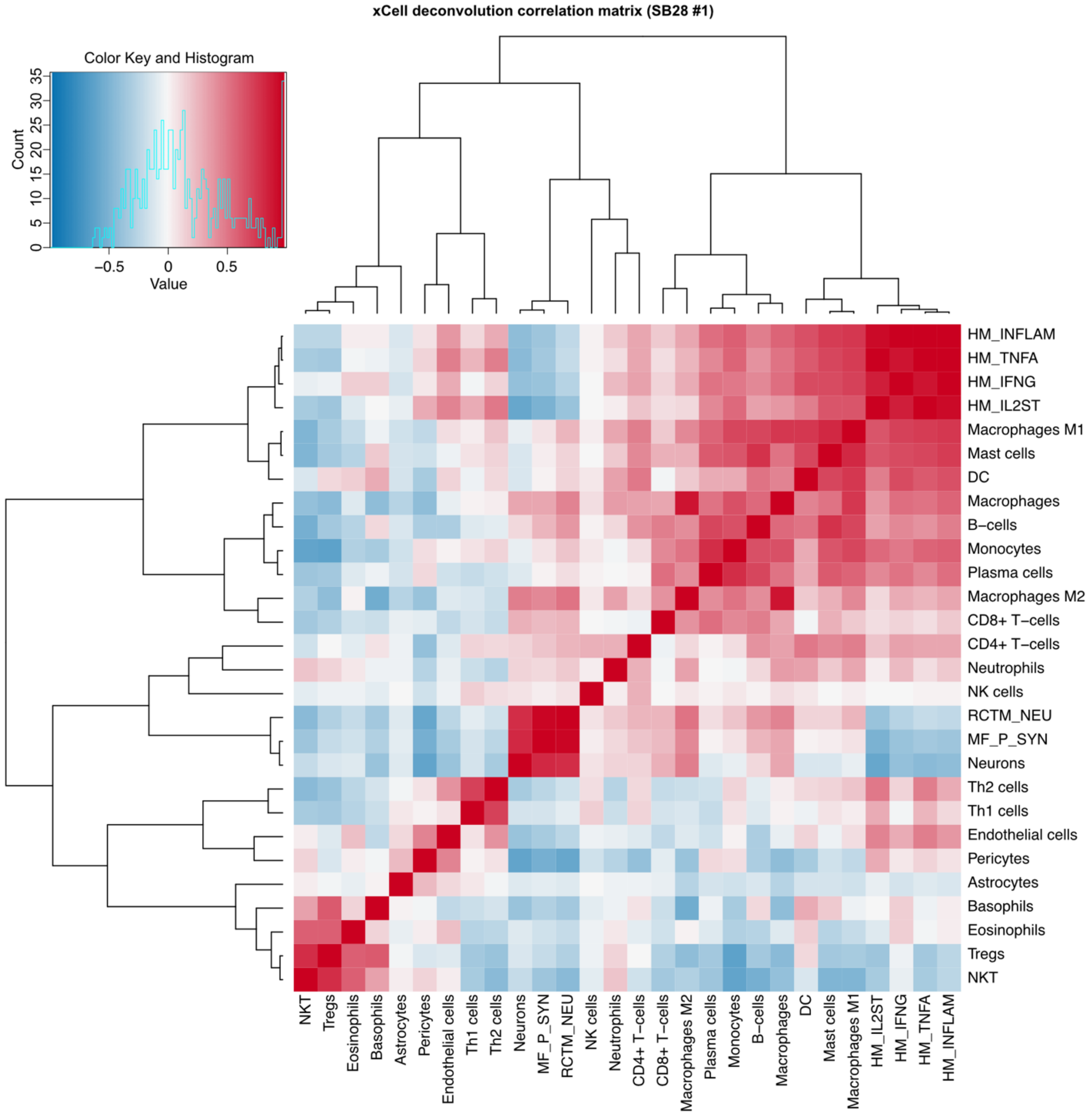

Supplementary Fig. 8 | Hierarchical clustering heatmap of pairwise correlations among the estimated cell abundances and gene sets. (Related to Fig. 2)
Cell-type deconvolution analysis performed on transcriptome data from each spot within the glioma-neuron infiltration area (n = 148 spots) using xCell[8]. Pearson correlations were calculated among the estimated scores for curated 22 cell types and 6 additional gene signature scores. Cell types and signature scores were reordered based on hierarchical clustering. In the heatmap, blue and red indicate positive and negative correlations, respectively.
Source data are provided as a Source Data file.

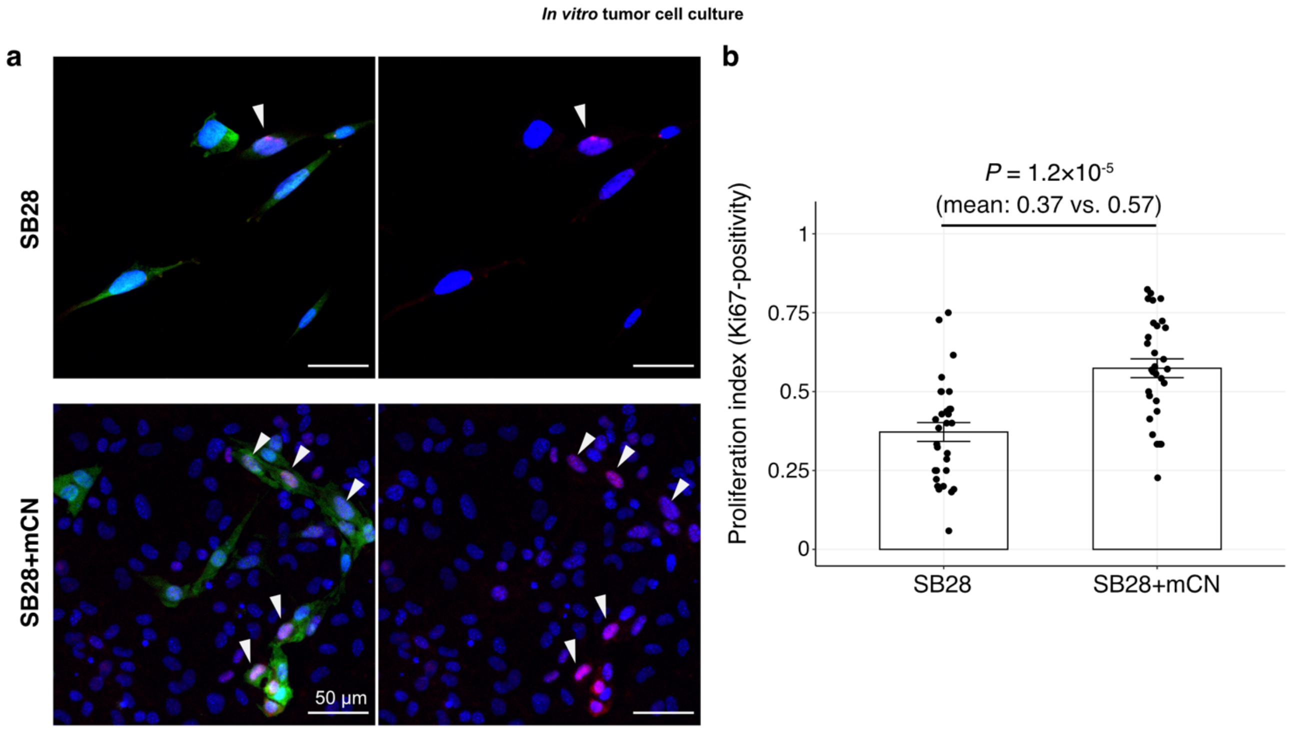

Supplementary Fig. 9 | Ki-67 proliferation index of SB28 with and without co-culture with neurons. (Related to Fig. 3)

a, Immunofluorescence images of SB28 cells cultured alone (top) or co-cultured with mouse cortical neurons (mCN) (bottom). Green: GFP-expressing SB28 cells; Red: Ki-67; Blue: DAPI. Scale bars, 50 µm.

b, Bar plot quantifying the proliferation index, calculated as the number of Ki67+ nuclei divided by the number of DAPI+ nuclei within GFP-expressing SB28 cells under each condition. Data were obtained from 30 randomly selected fields of view (FOVs) per group (663 µm × 663 µm each), across three independent culture experiments (10 FOVs per group per experiment). Mean proliferation index: 0.37 (SB28) vs. 0.57 (SB28+mCN) (Welch's unpaired *t*-test, $P = 1.2\times10^{-5}$). Data are presented as mean ± s.e.m. Source data are provided as a Source Data file.

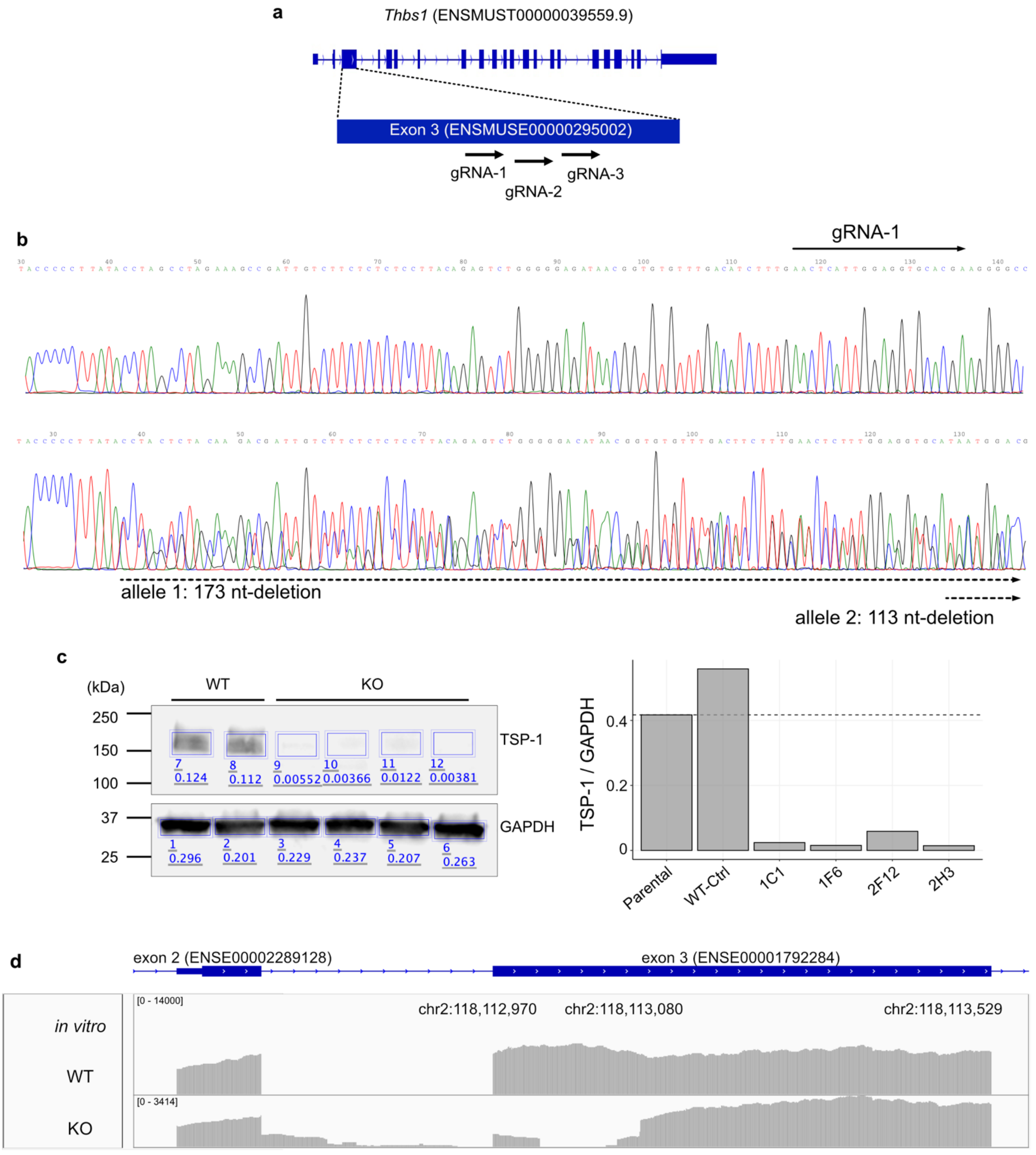

Supplementary Fig. 10 | CRISPR-KO of TSP1 in the SB28 mouse glioblastoma cell line. (Related to Fig. 3)

a, Schematic illustrating the design of the Gene KO kit v2 (Synthego) containing three multi-guide sgRNAs targeting exon 3 (ENSMUSE00000295002) of the murine TSP1 (*Thbs1*) gene.

b, Sanger sequencing results showing the *Thbs1* gene status in genomic DNA extracted from SB28-TSP1-WT (top) and KO clone 1C1 (bottom).

c, Western blot image with the regions of interest (ROIs) where signal intensity was measured. The accompanying bar plot shows the TSP1-to-GAPDH signal ratio. Signal values were calculated as the

total pixel intensity (Total) minus the product of Background and Area (Signal = Total - [Background × Area]). The uncropped blot image is provided in a Source Data file.
d, Snapshots from the Integrative Genome Viewer (IGV) showing exons 2–3 of the *Thbs1* gene and the mapped RNA-seq reads for SB28-TSP1-WT (top) and KO (bottom) in cultured cells.

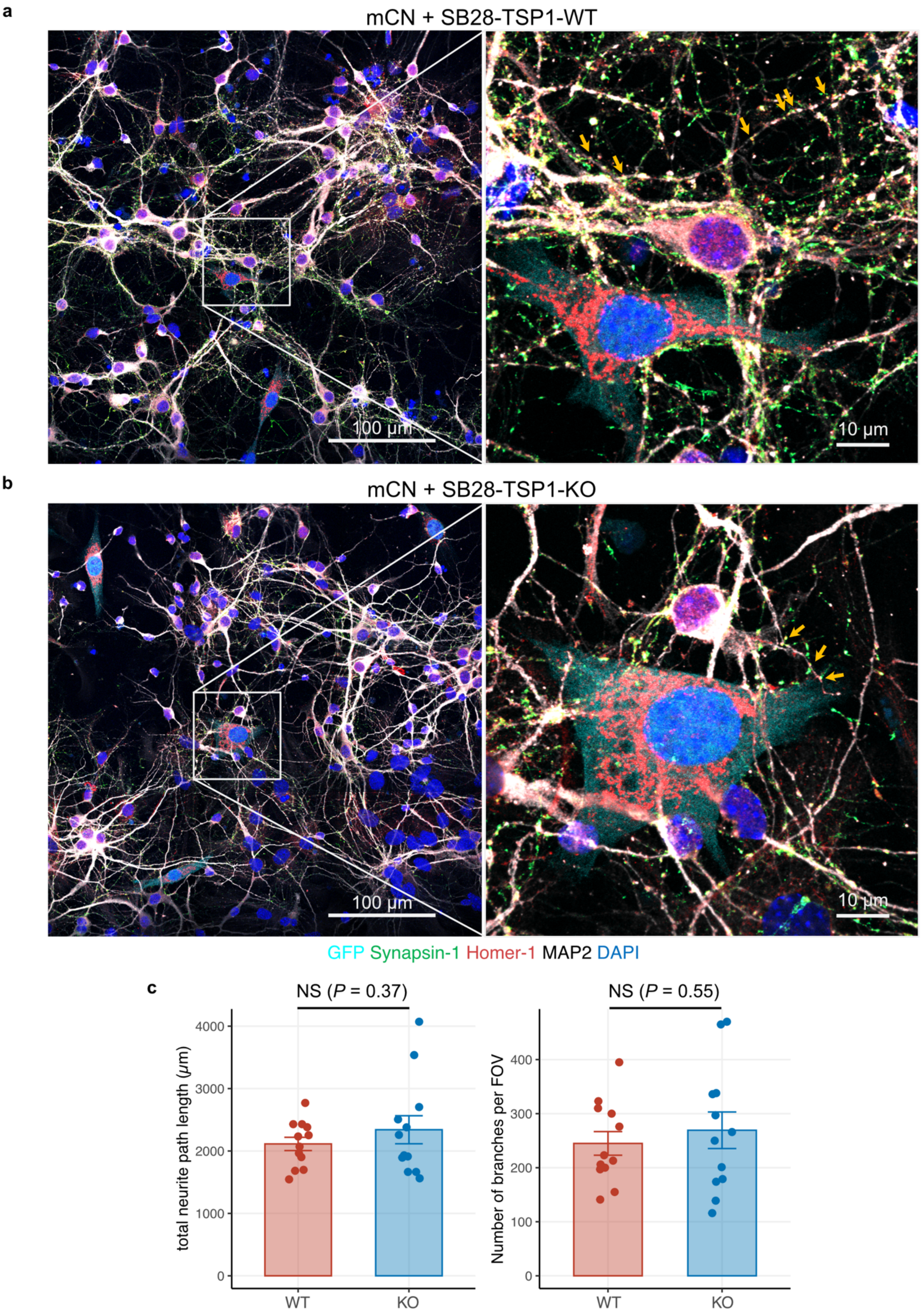

Supplementary Fig. 11 | Confocal image analysis of mouse cortical neurons co-cultured with SB28-TSP1-WT or KO cells. (Related to Fig. 3)

a, b, Representative confocal images showing synaptic punctum colocalization (yellow arrows) in co-cultures of murine neonatal cortical neurons with SB28-TSP1-WT (**a**) or KO (**b**) tumor cells, displayed at low magnification (405 µm × 405 µm, left) and high magnification (78 µm × 78 µm, right). High-magnification images correspond to those in **Fig. 4b**. Red, Homer-1; green, Synapsin-1; white, microtubule-associated protein 2 (MAP2, neurons); blue, 4',6-diamidino-2-phenylindole (DAPI); cyan, green fluorescent protein (GFP, SB28 tumors). Scale bars, 100 µm (left) and 10 µm (right).
c, Bar plots showing total neurite path length (µm) (left) and total number of branches (right) identified through skeletonized neurite images per 135 µm ×135 µm field of view (FOV) (n = 12 total FOVs per group, from 3 independent culture experiments).
P values were calculated using Welch's unpaired *t*-test. NS, not significant. Source data are provided as a Source Data file.

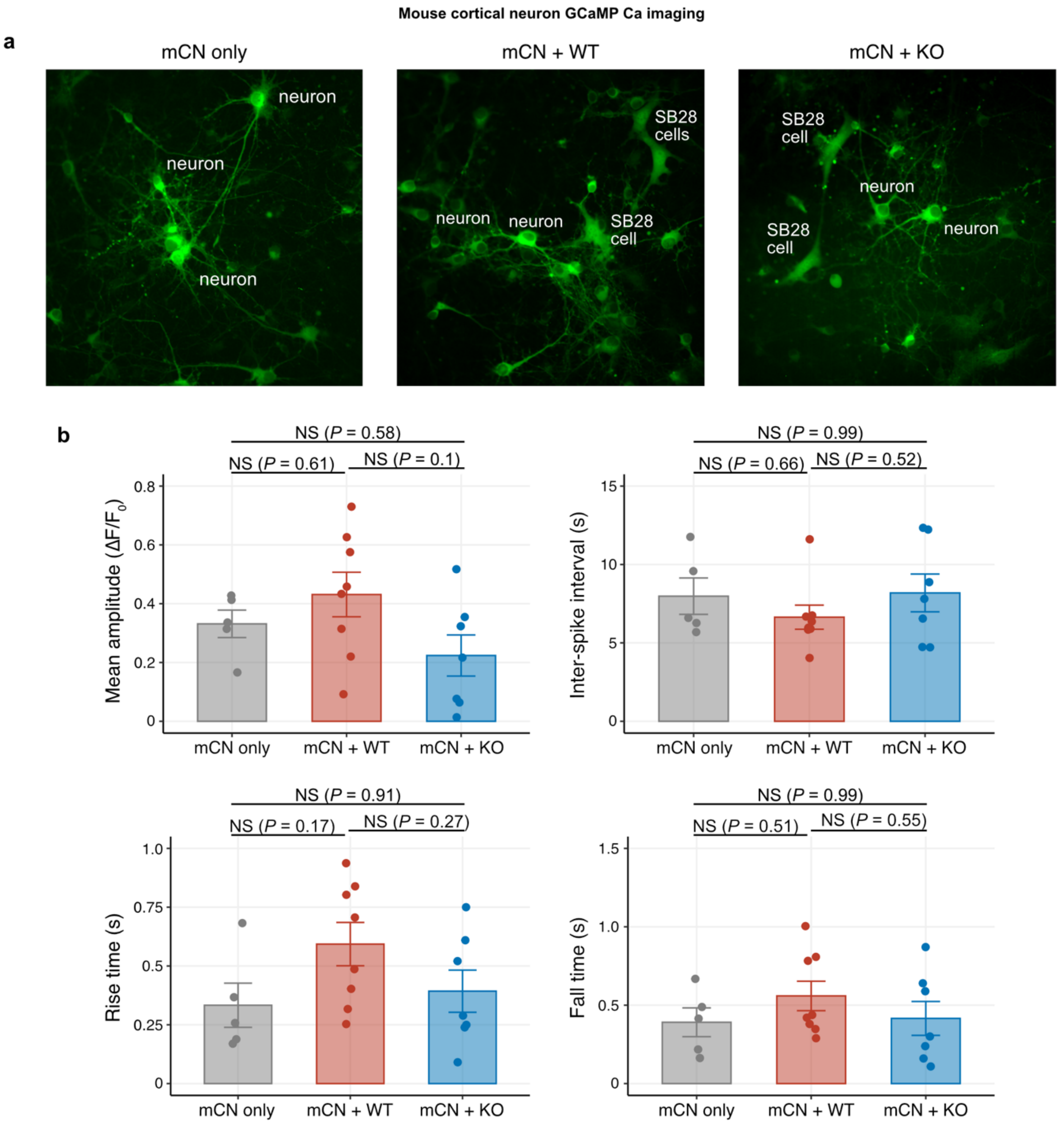

Supplementary Fig. 12 | Calcium signaling recordings of mouse cortical neurons. (Related to Fig. 3)

a, Representative micrographs showing GCaMP-positive neurons from three conditions: neurons alone ("mCN only," left), neurons co-cultured with SB28-TSP1-WT cells ("mCN + WT," middle), and neurons co-cultured with SB28-TSP1-KO cells ("mCN + KO," right). SB28 cells in the neuron-glioma co-culture were distinguished based on their morphology and constitutive GFP expression. Corresponding videos are available as **Supplementary Movies 1–3**.

b, Bar plots summarizing the quantification of single-cell properties of neurons, including the mean amplitude of calcium current (top-left), inter-spike interval (top-right), rise time (bottom-left) and and fall time (bottom-right) during the 5-minute calcium recording (n = 5 neurons [ROIs] for mCN only, 8 neurons for mCN + WT, and 7 neurons for mCN + KO).

P values were calculated using one-way analysis of variance (ANOVA) with Tukey's post hoc testing.

NS, not significant. Source data are provided as a Source Data file.

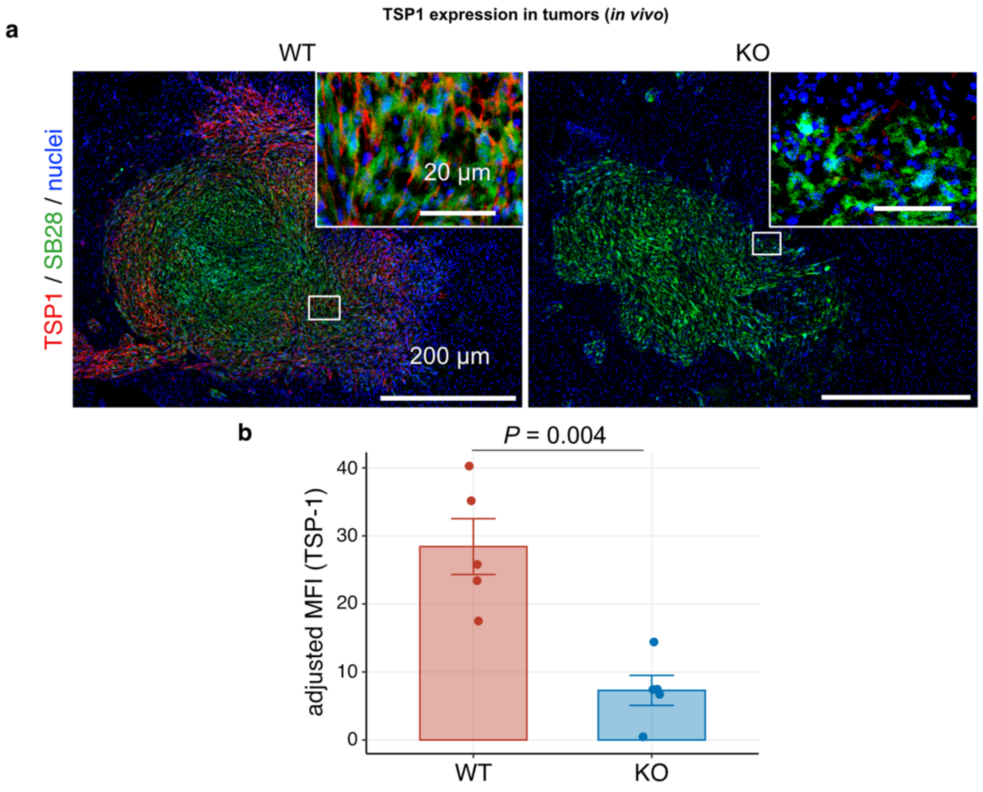

Supplementary Fig. 13 | TSP1 expression in SB28-TSP1-WT or KO *in vivo* tumors. (Related to Fig. 3)

a, Representative immunofluorescence images of tumor lesions in syngeneic mouse brains inoculated with SB28-TSP1-WT (left) and KO (right). Red, TSP1; green, GFP (SB28 tumor cells). Scale bars: 200 µm (low magnification) and 20 µm (high magnification).

b, Bar plot showing the adjusted mean fluorescence intensity (MFI) of TSP1 signals measured in the GFP-positive tumor area, adjusted for background fluorescence from the same tissue images (n = 5 mice per group). Data are presented as mean ± s.e.m.

P value was calculated using Welch's unpaired *t*-test. Source data are provided as a Source Data file.

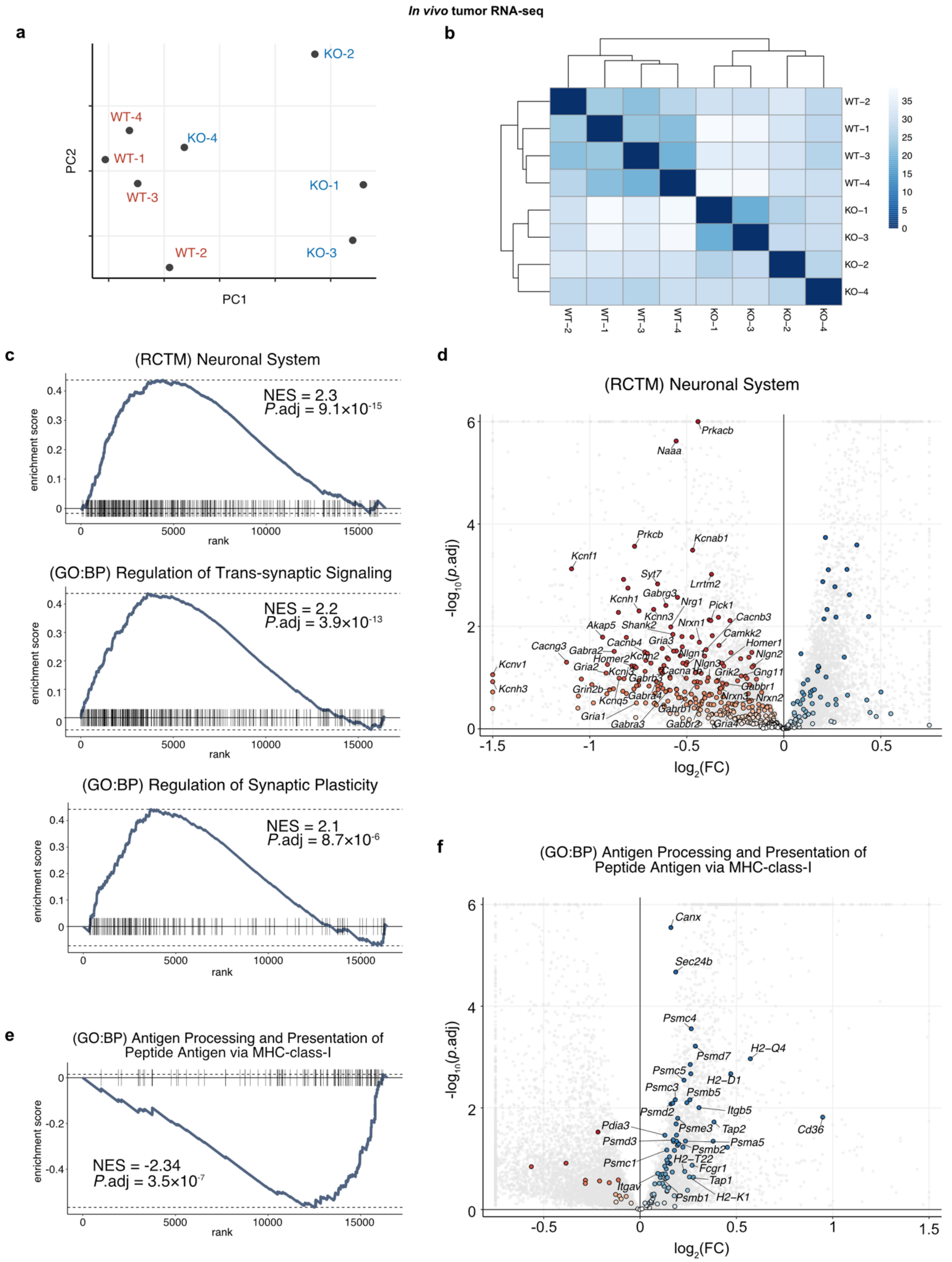

Supplementary Fig. 14 | Bulk RNA-seq of in vivo SB28-TSP1-WT and KO tumors. (Related to Fig. 3)

a, Principal component analysis (PCA) plot showing transcriptomic similarities and differences between i*n vivo* tumor samples from SB28-TSP1-WT (red) and KO (blue) groups (n = 4 mice per group).
b, Heatmap displaying hierarchical clustering of samples based on the most variably expressed genes, with sample-to-sample Euclidean distances calculated for each pair of samples.
c, Enrichment plots summarizing Gene Set Enrichment Analysis (GSEA) for the gene sets *Neuronal System* (Reactome), *Regulation of Trans-synaptic Signaling* (GO:BP), *Regulation of Synaptic Plasticity* (GO:BP), comparing gene expression profiles between SB28-TSP1-WT and KO mouse tumors. Positive normalized enrichment scores (NES) indicate upregulation in WT tumors compared to their KO counterparts.
d, Volcano plots showing the genes composing the *Neuronal System* (Reactome) gene set.
e, Enrichment plots summarizing GSEA for the gene set *Antigen Processing and Presentation of Peptide Antigen via MHC class I* (GO:BP), comparing gene expression profiles between SB28-TSP1-WT and KO mouse tumors. Negative NES indicates downregulation in WT tumors compared to their KO counterparts.
f, Volcano plots showing the genes composing *Antigen Processing and Presentation of Peptide Antigen via MHC class I* (GO:BP) gene set.
In volcano plots, genes in each gene set are highlighted in colors, with representative leading-edge gene symbols labeled. Genes with $\log_2$ fold change ($\log_2$FC) values and adjusted *P* values exceeding the boundaries are flattened and shown at the edges. Source data are provided as a Source Data file.

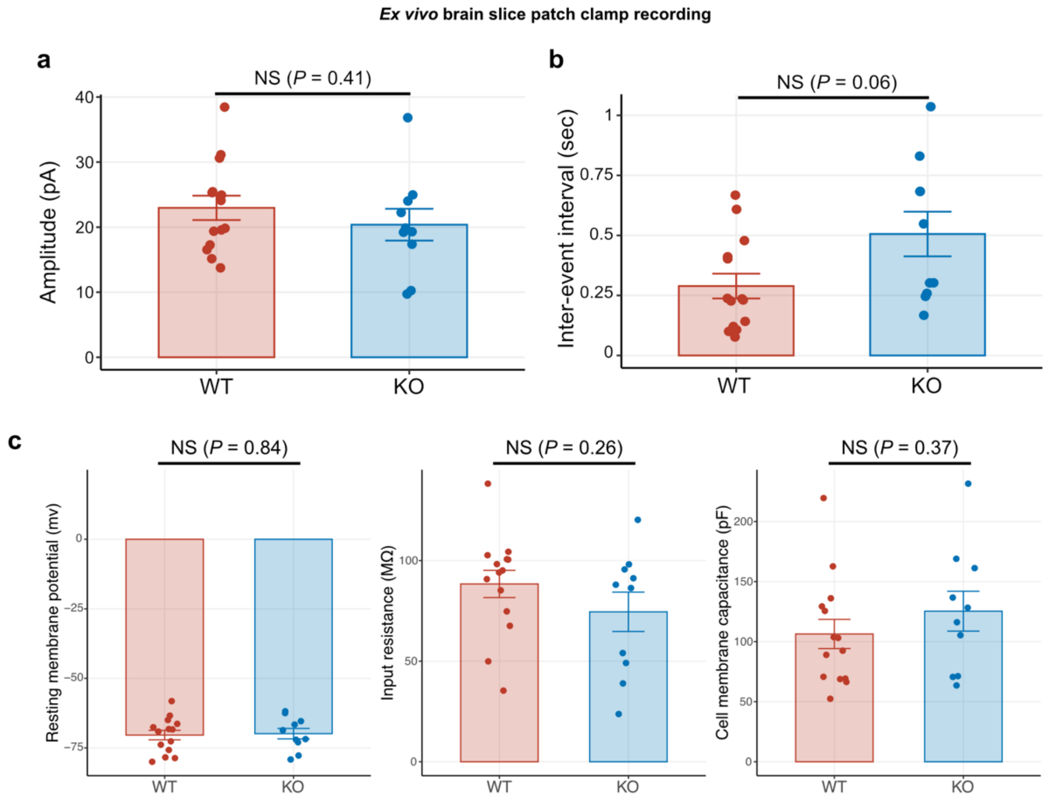

Supplementary Fig. 15 | *Ex vivo* mouse brain slice whole-cell patch clamp recordings (Related to Fig. 3)
a, b, Bar plot showing the amplitudes (**a**) and inter-event intervals (IEIs) (**b**) of spontaneous excitatory postsynaptic currents (EPSCs) measured simultaneously with the data presented in **Fig. 3g**

(n = 14 cells from 4 mice [WT] and 10 cells from 3 mice [KO]). Each dot represents the average value for an individual recorded cell.

c, Bar plots showing the following intrinsic property metrics: resting membrane potential (mean ± s.e.m. [mV]: -70.4 ± 1.71 [WT] and -69.9 ± 1.88 [KO], *P* = 0.84) (left); input resistance (mean ± s.e.m. [MΩ]: 88.4 ± 6.77 [WT] and 74.5 ± 9.78 [KO], *P* = 0.26) (middle); cell membrane capacitance (mean ± s.e.m [pF]: 106.4 ± 12.1 [WT] and 125.4 ± 16.6 [KO], *P* = 0.37) (right).
Data are presented as mean ± s.e.m.
P values were calculated using Welch's unpaired *t*-test.
NS, not significant. Source data are provided as a Source Data file.

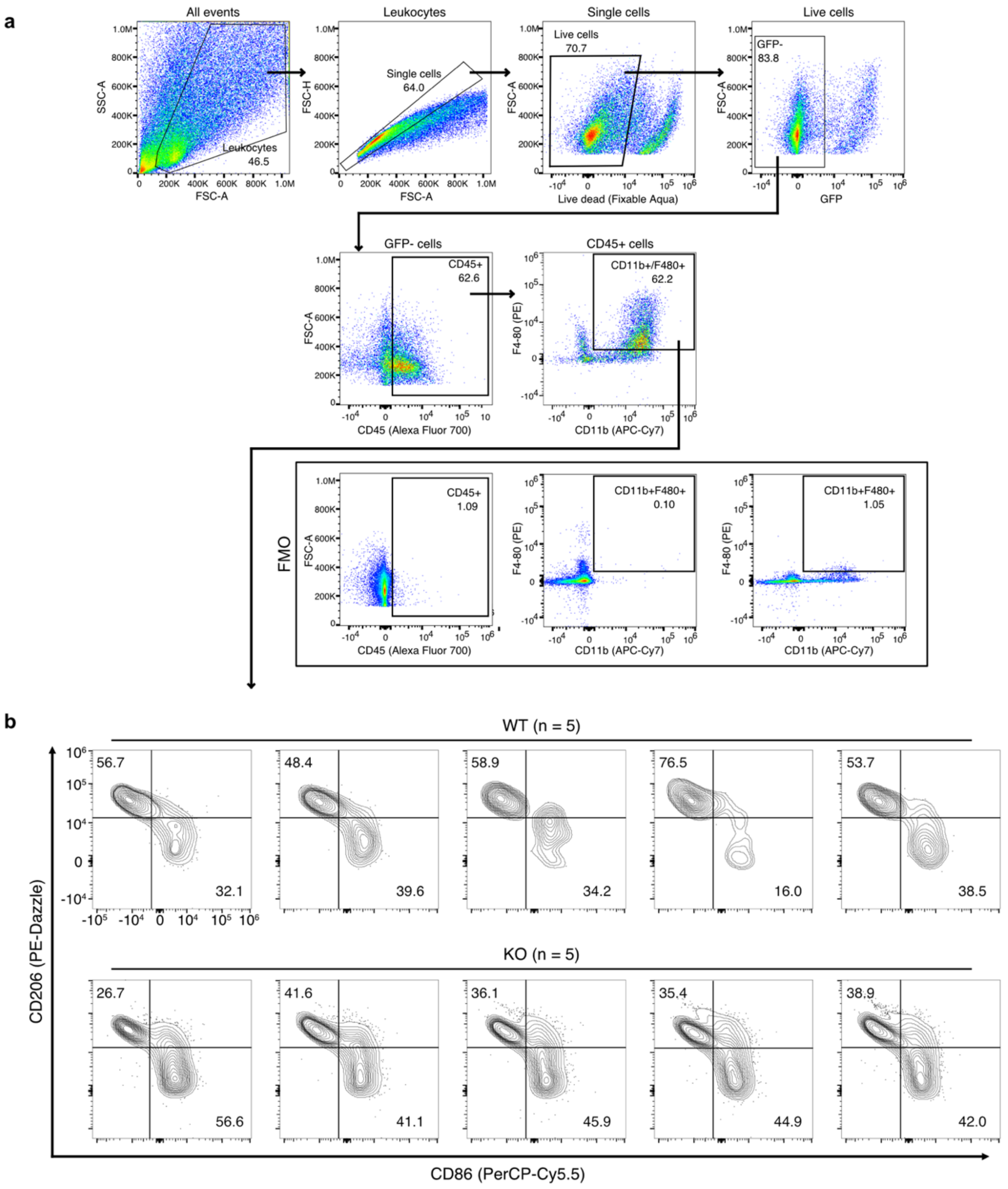

Supplementary Fig. 16 | Flow cytometry with TAM marker panel to characterize BILs isolated from SB28-TSP1-WT and KO tumors (Related to Fig. 4)

a, Gating strategy for flow cytometry to identify singlet/live/GFP-/CD45+/CD11b+/F480+ cell populations (defined as TAMs) within brain infiltrating leukocytes (BILs).

b, Contour plots showing the expression of CD86 and CD206 on TAMs isolated from individual mouse brains bearing SB28-TSP1-WT or KO tumors (n = 5 mice per group). Values in the plots represent the percentages of the cell populations within the gates.

FMO, fluorescence minus one.

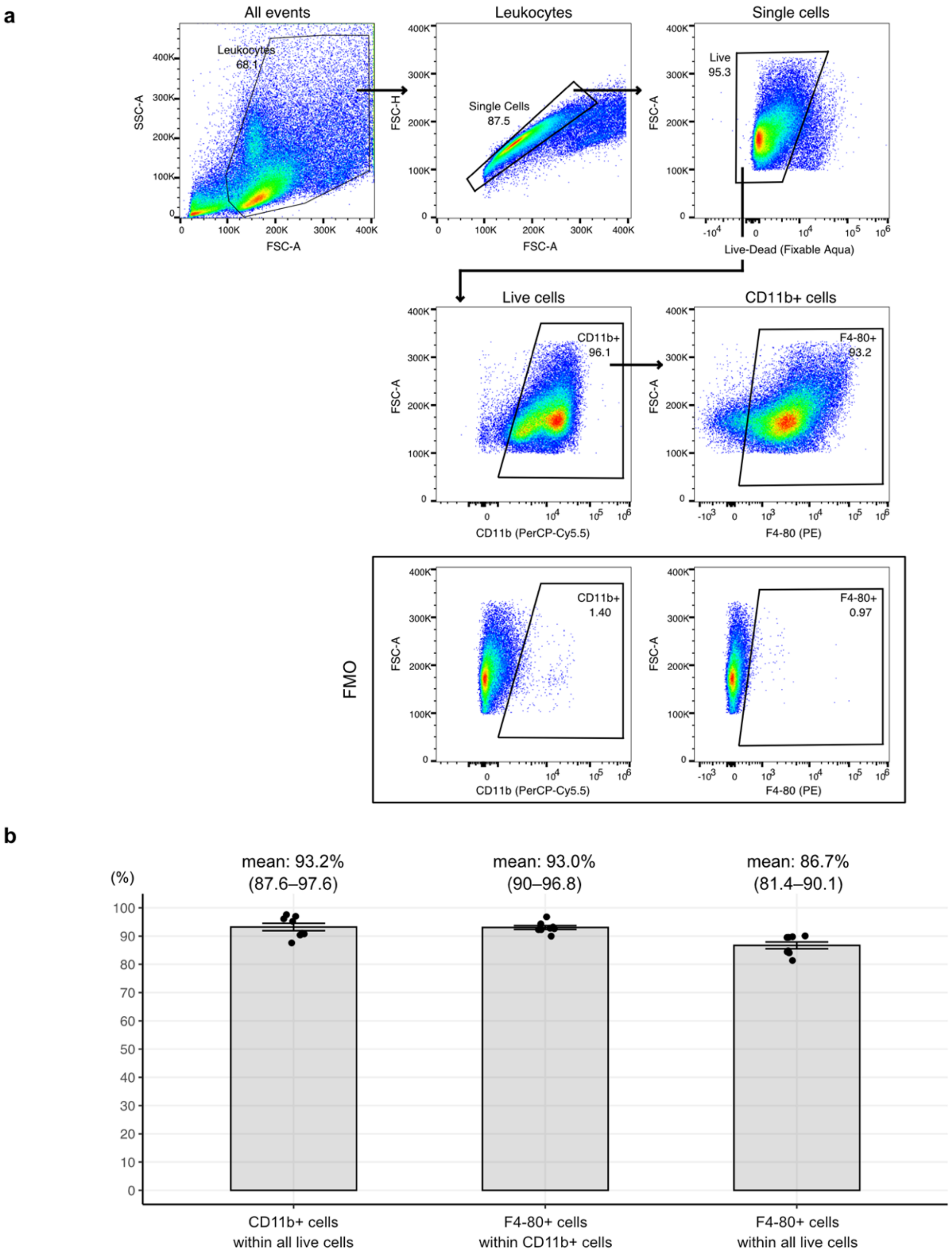

Supplementary Fig. 17 | Characterization of magnetic bead-sorted CD11b+ BILs (Related to Fig. 4)

a, Gating strategy for flow cytometry to identify singlet/live/CD11b+/F4-80+ cell populations within CD11b+ magnetic bead-sorted BILs.

b, Bar plots showing the percentages of CD11b+ cells within all live cells (left), F480+ cells within CD11b+ cells (middle), and F4-80+ cells within all live cells (right). Data are presented as mean ± s.e.m.

FMO, fluorescence minus one. Source data are provided as a Source Data file.

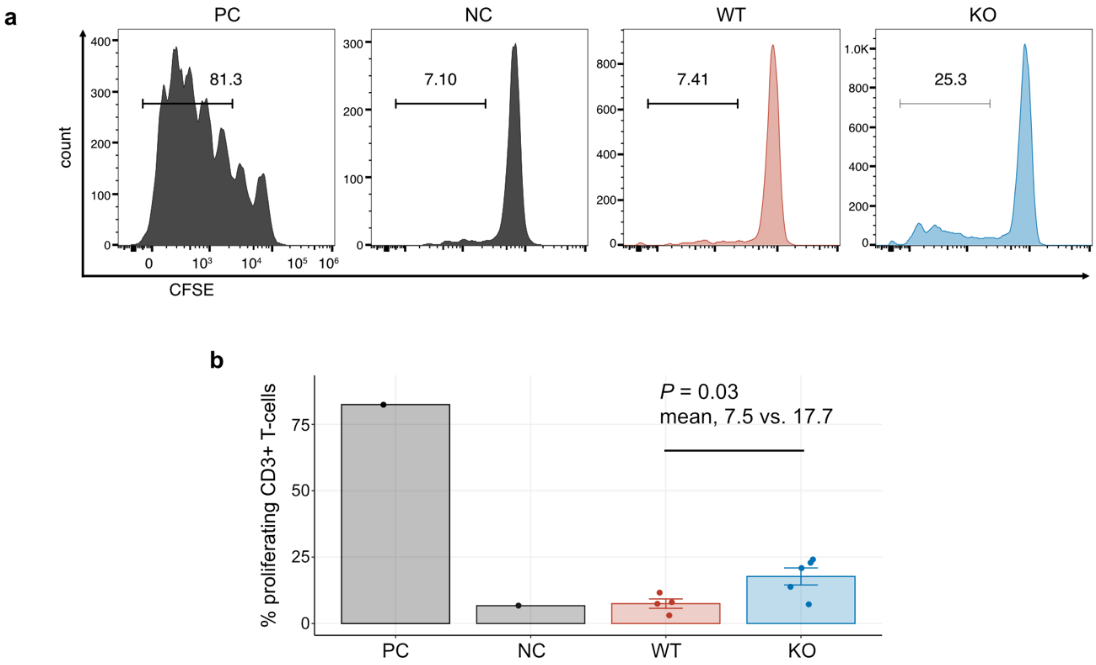

Supplementary Fig. 18 | Immunosuppression assay of WT- or KO-tumor-derived TAMs (Related to Fig. 4)

a, Flow cytometry histogram showing the peak distributions of CFSE signals in positive (PC) and negative controls (NC), and representative samples from the WT and KO groups.

b, Bar plot displaying the percentages of proliferating CD3+ T-cells. CD11b+ BILs isolated from 4 WT-tumor-bearing mice and 5 KO tumor-bearing mice were evaluated.

P value was calculated using Welch's unpaired *t*-test. Data are mean ± s.e.m.

Source data are provided as a Source Data file.

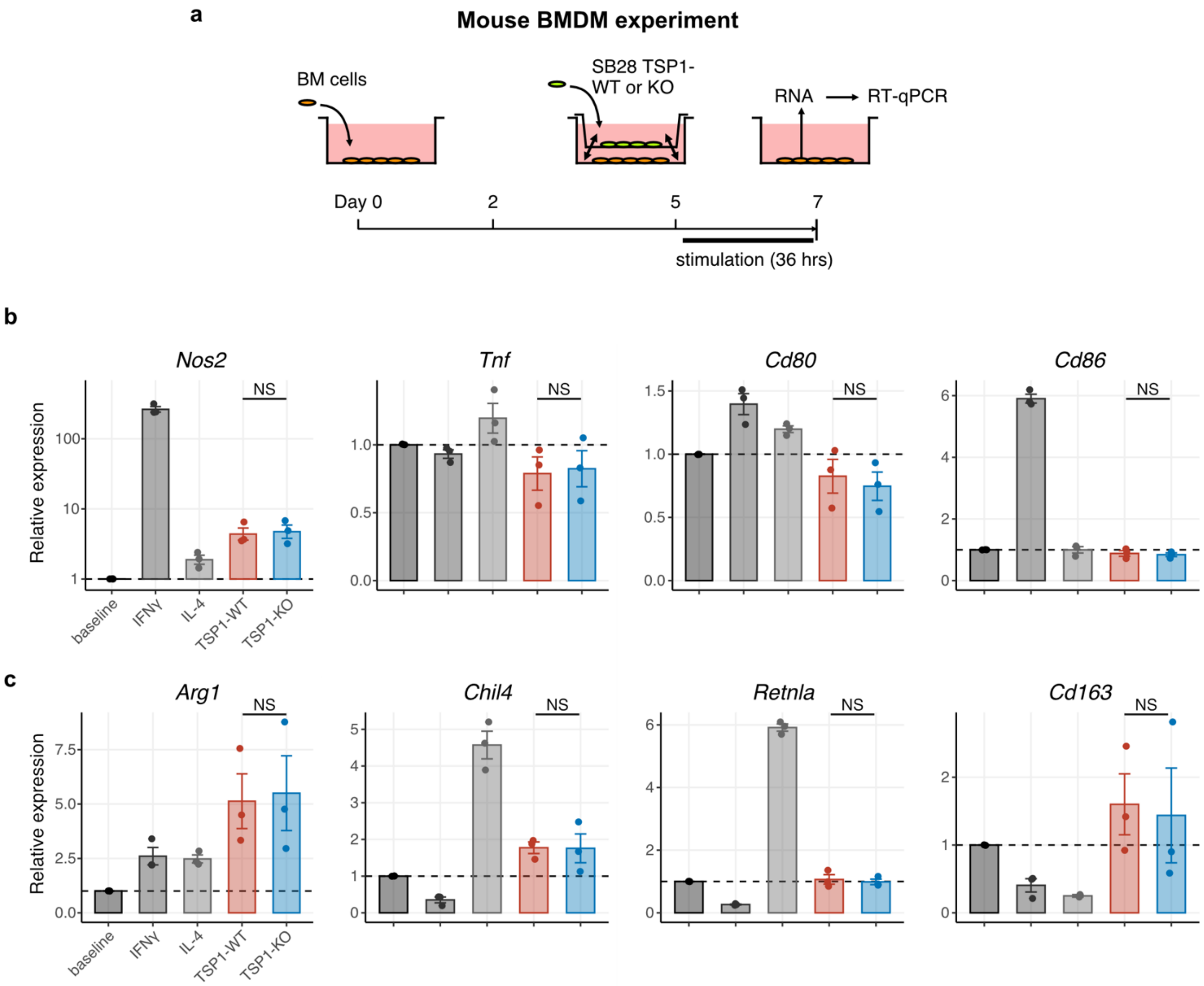

Supplementary Fig. 19 | Quantitative analysis of *ex vivo* BMDM reprogramming assay (Related to Fig. 4).

a, Schematic overview of *ex vivo* BMDM reprogramming experiment to assess the effects of SB28-TSP1-WT and KO cell co-culture. Mouse bone marrow (BM) cells were isolated from tibias and femurs of C57BL/6J mice (n = 3 individuals) and maintained with GM-CSF stimulation. From day five, BMDMs were co-cultured with SB28-TSP1-WT or KO cells using well inserts to allow the exchange of secreted factors while preventing direct contact of BMDMs and tumor cells. After 36 hours of co-culture, the tumor cell-containing inserts were removed, and total RNA was extracted from the BMDM compartment.

b, c, Bar plots showing RT-qPCR analysis of representative pro-inflammatory (**b**) and anti-inflammatory (**c**) marker expression. IFNγ and IL4 served as positive controls. Relative expression values were calculated using the $2^{-\Delta\Delta Cq}$ method.

P values were calculated using Welch's unpaired *t*-test. NS, not significant.

Source data are provided as a Source Data file.

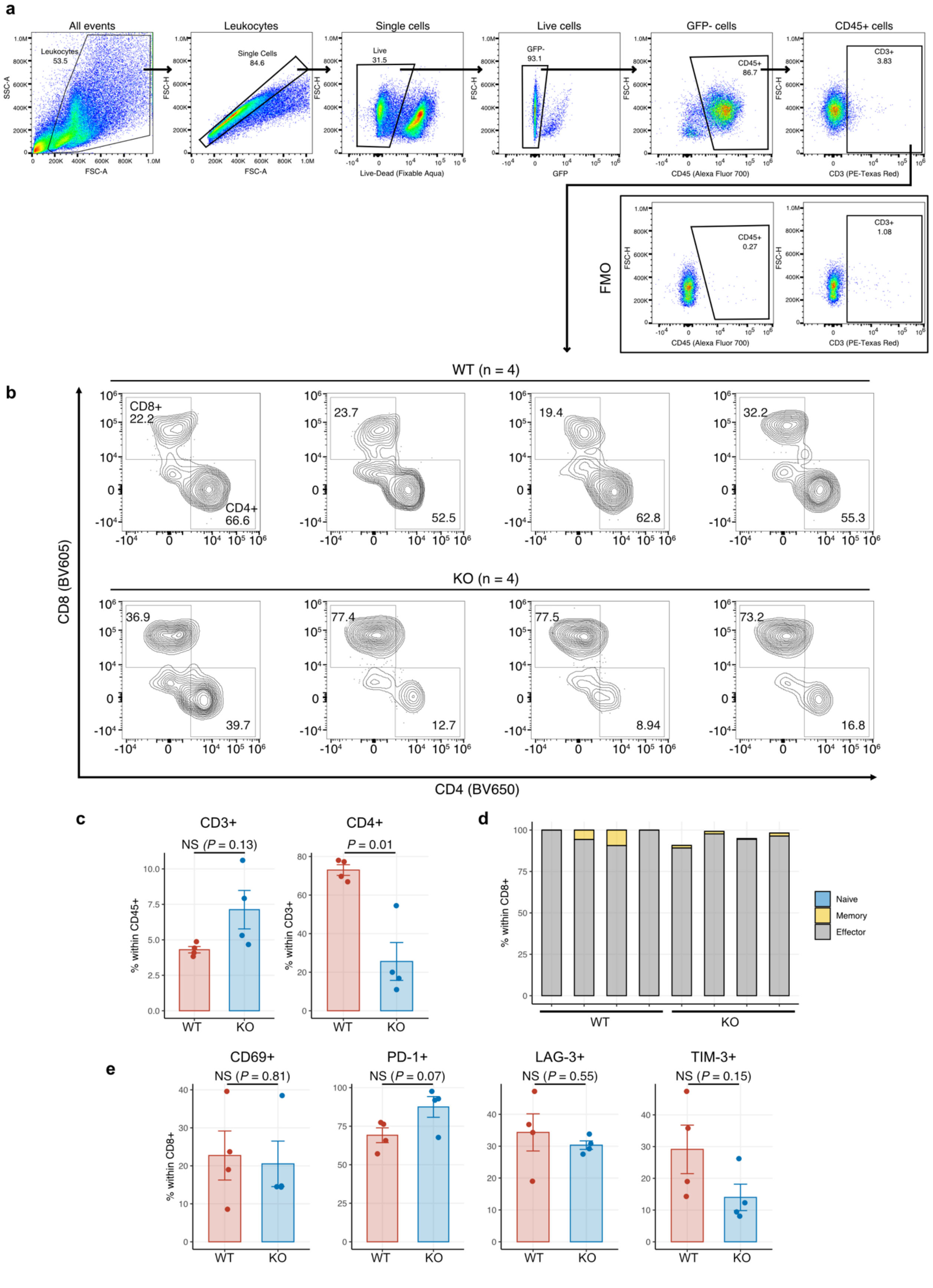

Supplementary Fig. 20 | Flow cytometry with T-cell marker panel to characterize BILs isolated from SB28-TSP1-WT and KO tumors (Related to Fig. 4)

a, Gating strategy for flow cytometry to identify singlet/live/GFP-/CD45+/CD3+ cell populations (defined as CD3+ T-cells) within brain infiltrating leukocytes (BILs).
b, Contour plots showing the expression of CD4 and CD8 on CD3+ T-cells isolated from individual mouse brains bearing SB28-TSP1-WT or KO tumors (n = 4 mice per group). Values in the plots represent the percentages of cell populations within the gates.
c, Bar plot showing the percentages of CD3+ T-cells within CD45+ cells (left) and CD4+ T-cells within CD3+ T-cells (right) (n = 4 mice per group; CD3+, $P = 0.13$, mean, 4.3% vs. 7.1%; CD4+, $P = 0.01$, mean, 73.0% vs. 25.6%).
d, Bar plots showing the percentages of naïve (blue), memory (yellow), and effector (gray) phenotypes within CD8+ T-cells in each sample.
e, Bar plots showing the percentages of CD8+ T-cells positive for activation or exhaustion markers. *P* values were calculated using Welch's unpaired *t*-test. FMO, fluorescence minus one. NS, not significant. Source data are provided as a Source Data file.

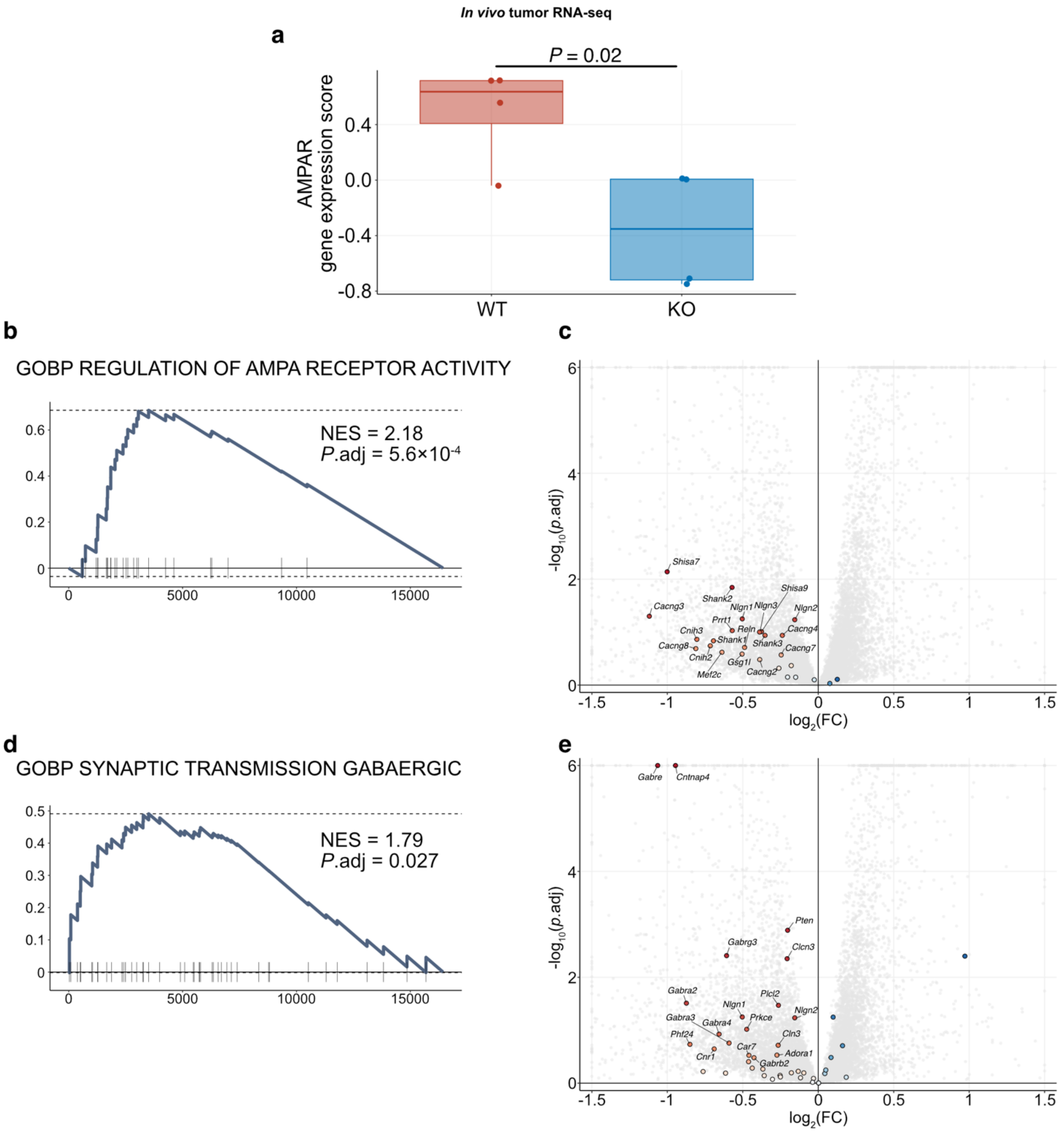

Supplementary Fig. 21 | Pathway analyses of AMPAR and GABA-related signals. (Related to Fig. 5)

a, Box plot showing the AMPA-R scores calculated based on bulk RNA-seq data of *in vivo* tumors with TSP1-WT and KO (n = 4 each).

b–e, Enrichment plots summarizing GSEA with the gene sets *Regulation of AMPA Receptor Activity* (GO:BP) (**b**), *Synaptic Transmission Gabaergic* (GO:BP) (**d**) to compare the gene expression patterns between the SB28-TSP1-WT and KO tumors. In the corresponding volcano plots (**c** and **e**), the genes composing the gene sets are highlighted in colors, and among them, the representative leading-edge gene symbols are labeled. Background gray dots are all the protein-coding genes in the dataset. Genes with log_2FC values and adjusted *P* values exceeding the boundaries are flattened and shown at the edges.

P value was calculated using Welch's unpaired *t*-test (**a**).

NES, normalized enrichment score; FC, fold change. Source data are provided as a Source Data file.

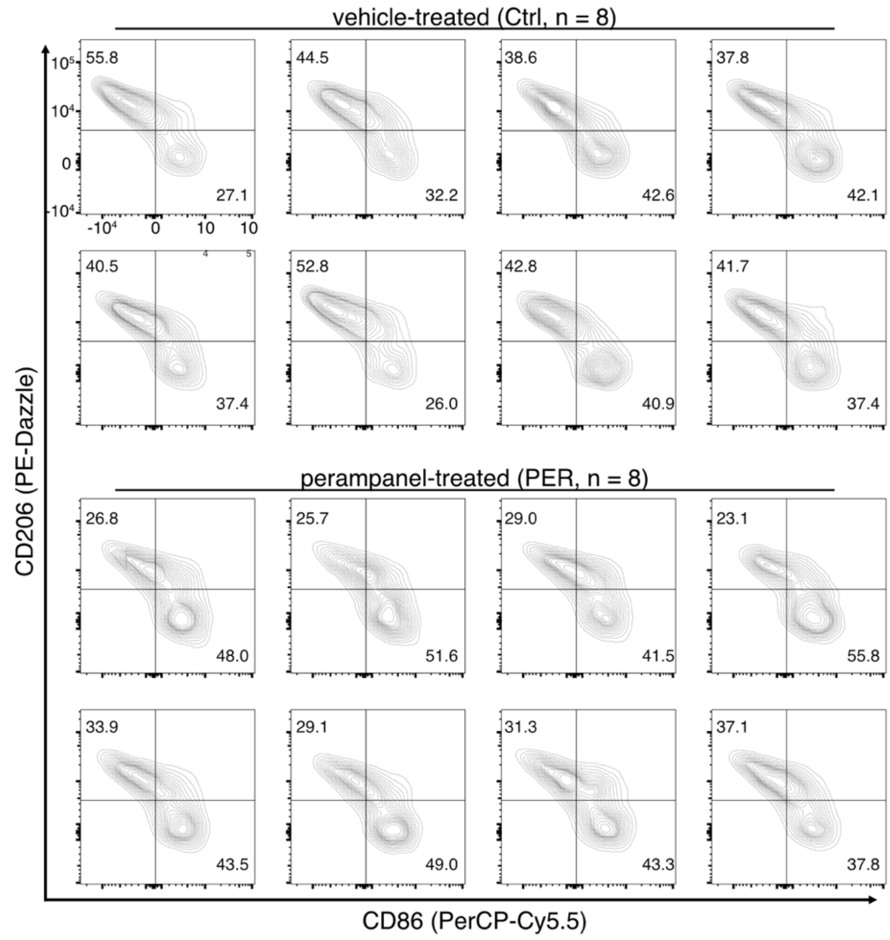

Supplementary Fig. 22 | Flow cytometry with TAM marker panel to characterize PER-treated and untreated tumor-bearing mouse brains (Related to Fig. 5)

Contour plots showing the expression of CD86 and CD206 on TAMs isolated from SB28-TSP1-WT tumor-bearing mice treated with vehicle (Ctrl) or perampanel (PER) (n = 8 mice per group). Values in the plots represent the percentages of cell populations within the gates.

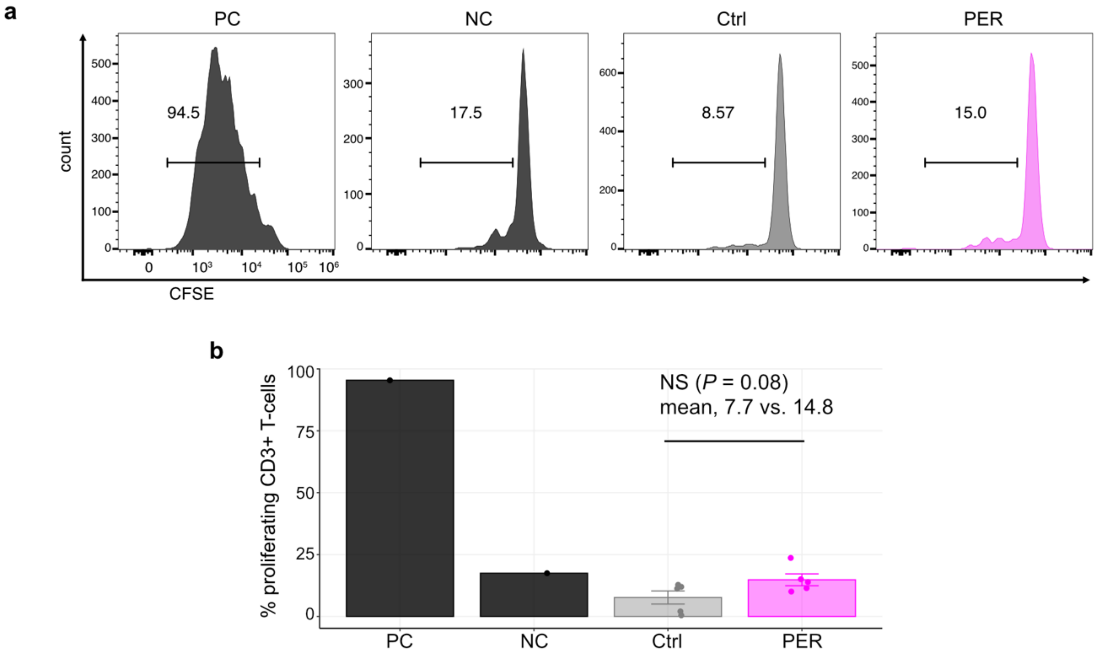

Supplementary Fig. 23 | Immunosuppression assay of TAMs from PER-treated and untreated mice (Related to Fig. 5)

a, Flow cytometry histogram showing the peak distributions of CFSE signals in positive (PC) and negative controls (NC), and representative samples from the untreated (Ctrl) and perampanel-treated (PER) groups.

b, Bar plot displaying the percentages of proliferating CD3+ T-cells. CD11b+ BILs isolated from 5 mice per group were evaluated. Data are mean ± s.e.m.

P value was calculated using Welch's unpaired *t*-test. NS, not significant. Source data are provided as a Source Data file.

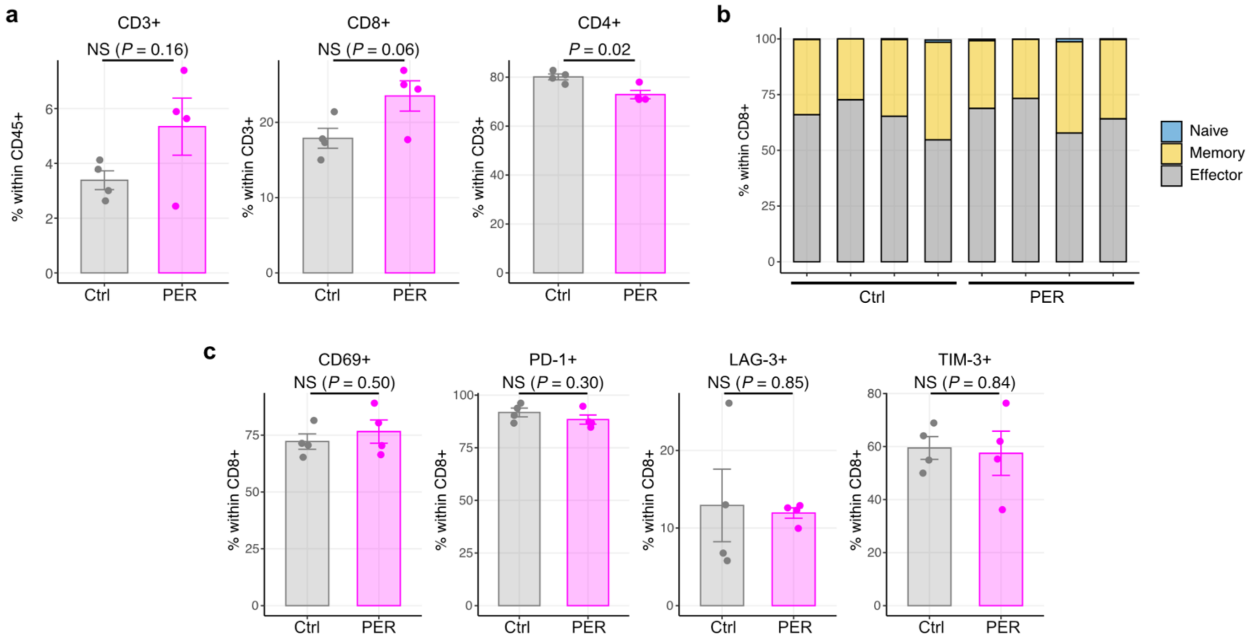

Supplementary Fig. 24 | Flow cytometry with T-cell marker panel to characterize PER-treated and untreated tumor-bearing mouse brains (Related to Fig. 5)

a, Bar plots showing the percentages of CD3+ T-cells within CD45+ cells (left), CD8+ T-cells within CD3+ T-cells (middle), and CD4+ T-cells within CD3+ T-cells (right) (n = 4 mice per group). CD3+ T-cells were defined as singlet/live/GFP-/CD45+/CD11b-/CD3+ cell population.

b, c, Bar plots showing the percentages of naïve (blue), memory (yellow), and effector (gray) phenotypes within CD8+ T-cells for each sample (**b**), and the percentages of CD8+ T-cells positive for activation or exhaustion markers (**c**).

P values were calculated using Welch's unpaired *t*-test. NS, not significant. Source data are provided as a Source Data file.

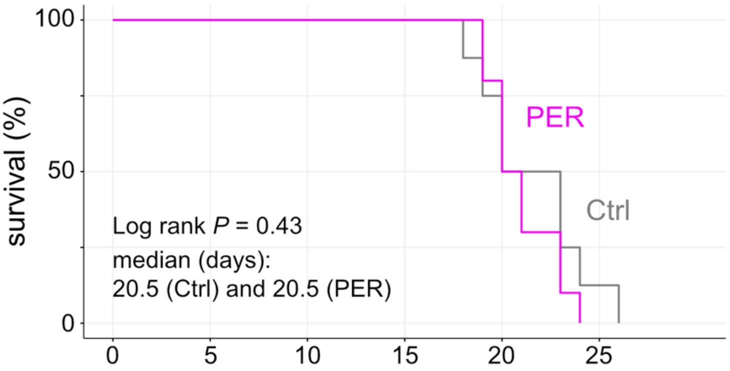

Supplementary Fig. 25 | *In vivo* tumor growth curves and survival trends comparing PER-treated and non-treated SB28-TSP1-KO tumor-bearing mouse. (Related to Fig. 5)

Kaplan-Meier survival curves of C57BL/6J mice orthotopically inoculated with SB28-TSP1-KO cells (10,000 cells/1 µL/mouse) and treated with perampanel (PER, n = 8 mice), or vehicle control (Ctrl, n = 10 mice), starting the day after tumor inoculation.

P value was calculated using Log-rank test. Source data are provided as a Source Data file.

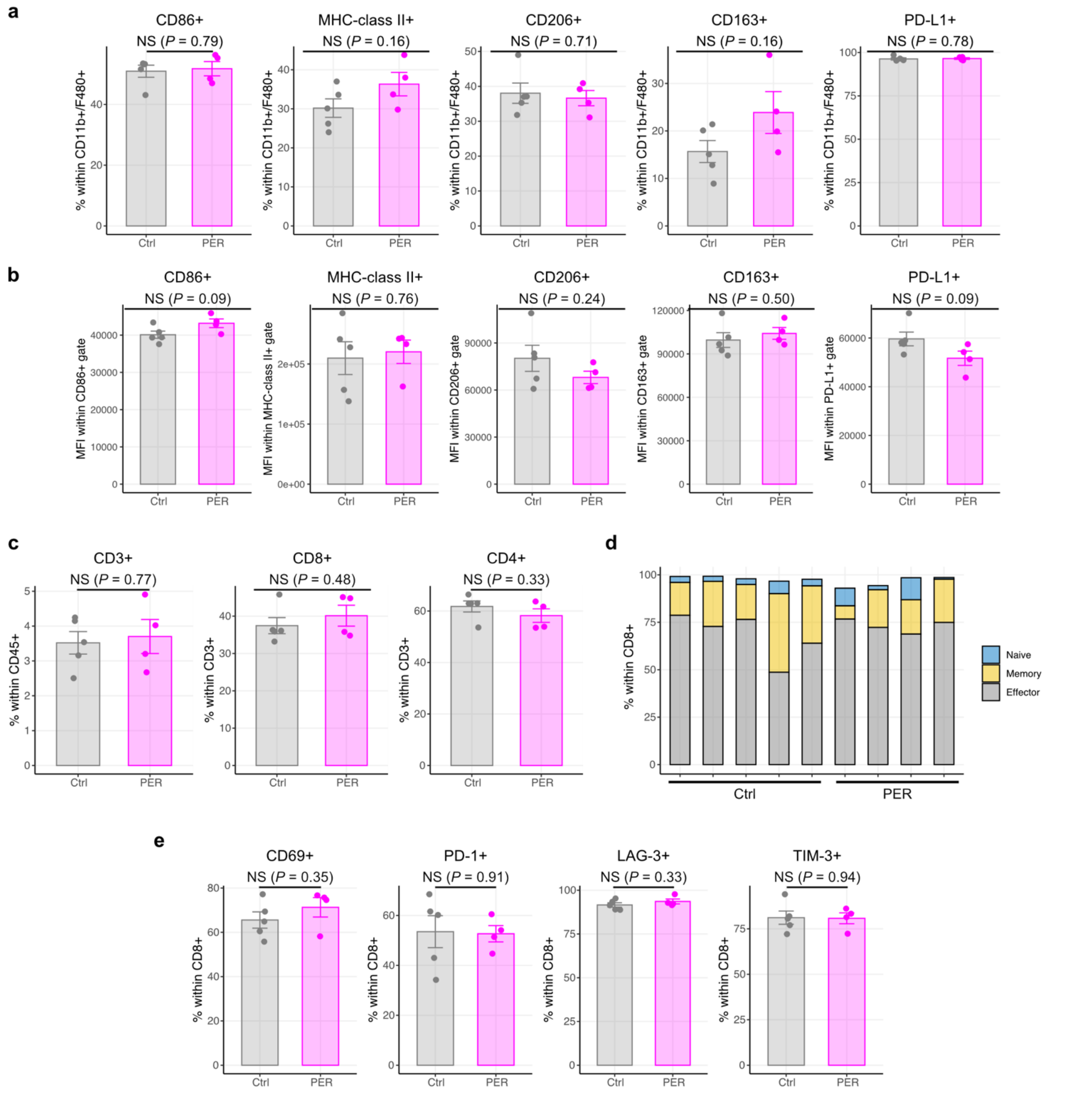

Supplementary Fig. 26 | Flow cytometry to characterize PER-treated and untreated mouse brains with SB28-TSP1-KO tumors (Related to Fig. 5)

Flow cytometry analysis of tumor-associated macrophage (TAM) (**a, b**) and T-cell (**c–e**) marker panels to characterize brain-infiltrating leukocytes (BILs) isolated from individual SB28-TSP1-KO tumor-bearing mice treated with vehicle control (Ctrl, n = 5) or perampanel (PER, n = 4). TAMs were defined as singlet/live/GFP-/CD45+/CD11b+/F480+ cell populations. CD3+ T-cells were defined as singlet/live/GFP-/CD45+/CD11b-/CD3+ cell populations.

a, b, Bar plots showing the percentages of CD86+, MHC-class-II+, CD206+, CD163+, and PD-L1+ cells within the TAM population (top), and their mean fluorescence intensities (MFIs) within each marker-positive population (bottom).

c, Bar plots showing the percentages of CD3+ T-cells within CD45+ cells (left), CD8+ T-cells within CD3+ T-cells (middle), and CD4+ T-cells within CD3+ T-cells (right).

d, e, Bar plots showing the percentages of naïve (blue), memory (yellow), and effector (gray) phenotypes within CD8+ T-cells for each sample (**d**), and the percentages of CD8+ T-cells positive for activation or exhaustion markers (**e**).
P values were calculated using Welch's unpaired *t*-test. NS, not significant. Source data are provided as a Source Data file.

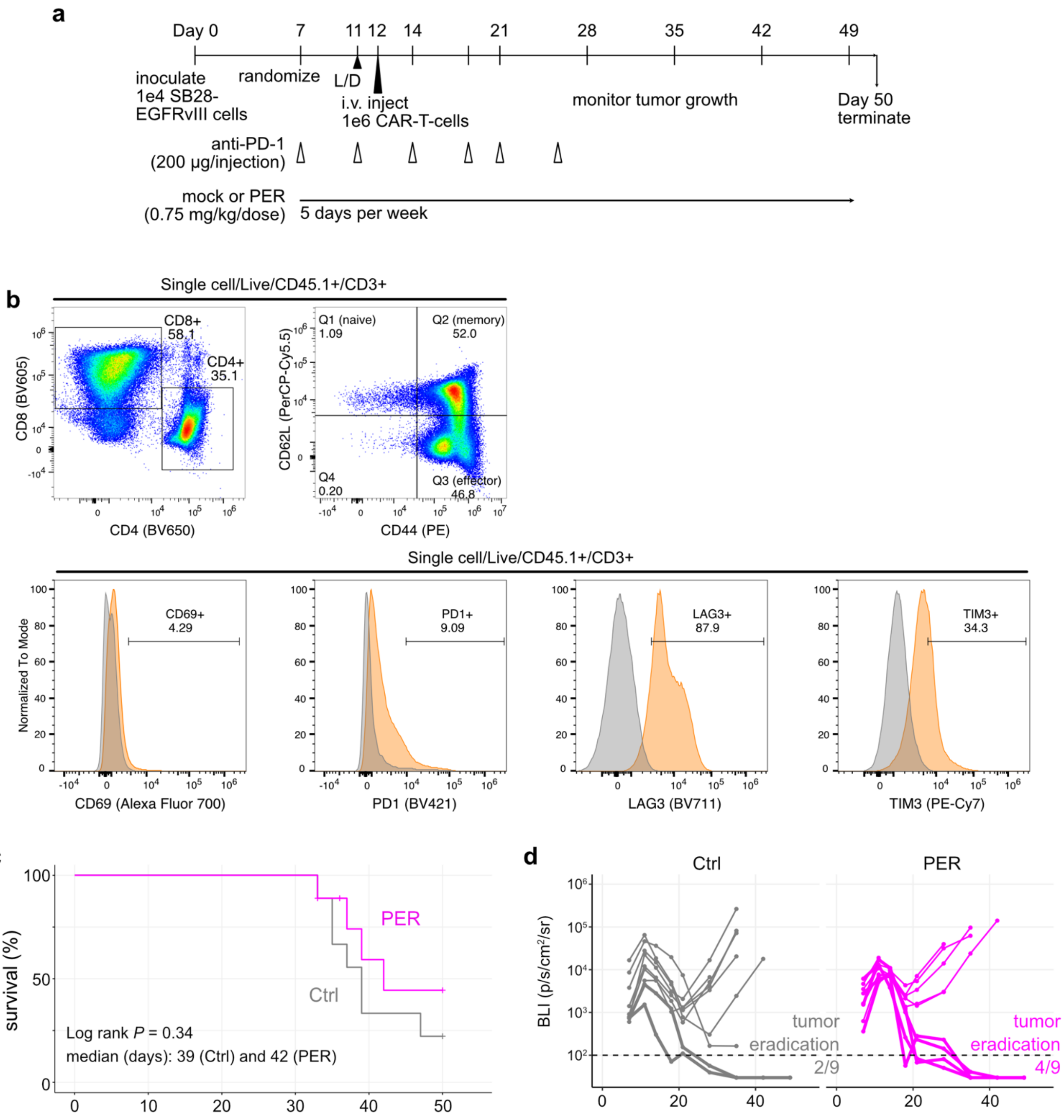

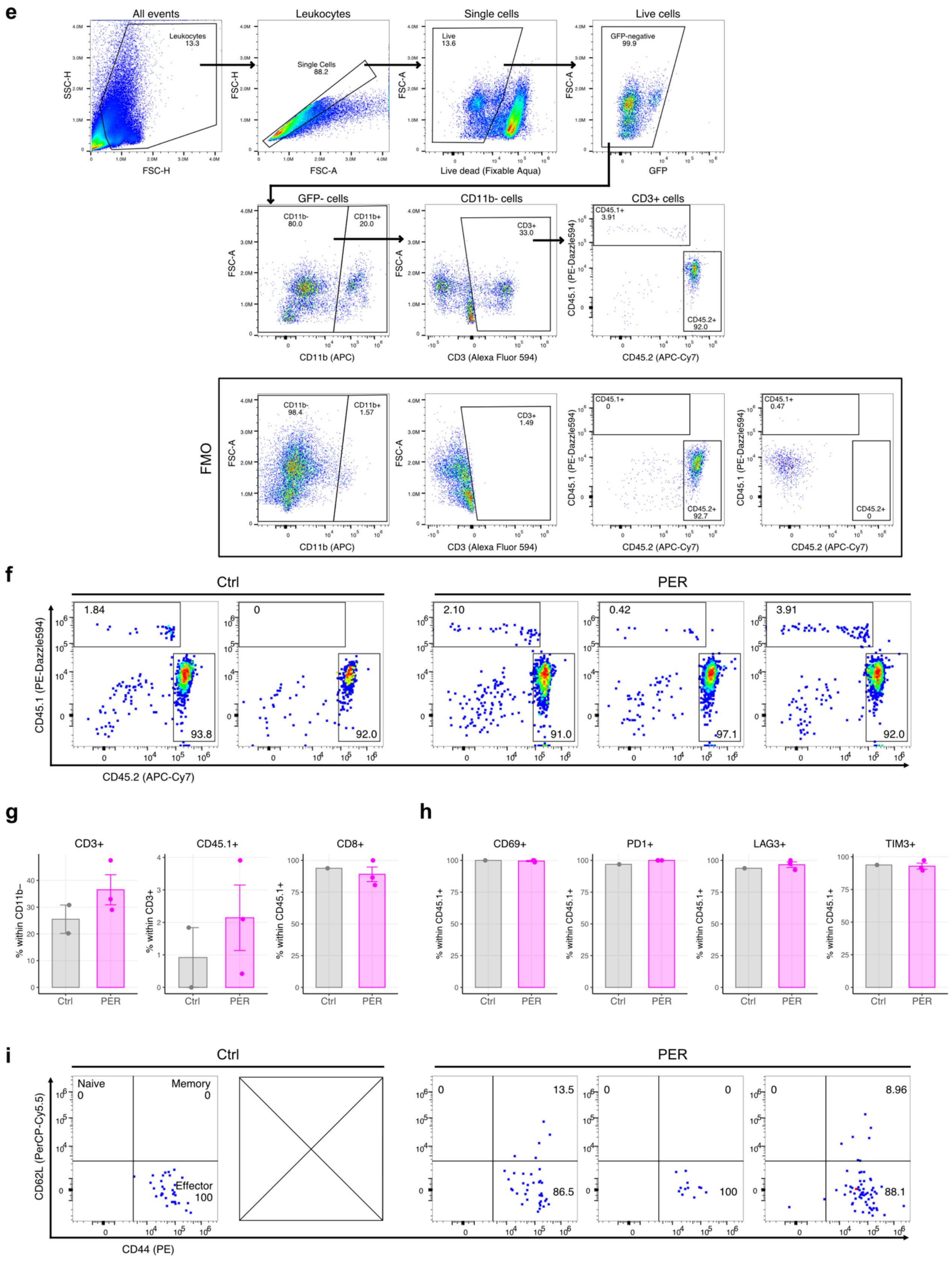

Supplementary Fig. 27 | *In vivo* study investigating the combination immunotherapy of CAR T-cells and anti-PD-1 antibody with and without PER (Related to Fig. 5)

a, Schematic illustration of experimental design. On day 7 after orthotopic inoculation of SB28-TSP1-WT, EGFRvIII+ tumor cells, mice were assigned to either the PER-treated or mock-treated (Ctrl) group based on bioluminescence imaging (BLI) signals (n = 9 mice each). Oral gavage of PER was administered five days per week starting on day 7 until the endpoint of each individual. Anti-PD-1 antibody was administered intraperitoneally twice a week starting on day 7, for a total of six doses, as previously reported [9]. Anti-EGFRvIII-CAR T-cells were isolated from a CAR T transgenic mouse, and subsequent priming was performed as previously reported[10]. Systemic lymphodepletion was achieved using cyclophosphamide and fludarabine on day 11, followed by trans-retroorbital infusion of 1×10^6 CAR T-cells on day 12. Mice were monitored via BLI once or twice a week and euthanized based on predefined endpoint criteria. On day 50 post-inoculation, all surviving mice were euthanized after confirming complete tumor eradication.
b, Flow cytometry scatter plots and histograms showing the characteristics of CAR T-cells after a 3-day priming period (at the time of infusion), including T-cell lineage, differentiation status, and activation and exhaustion marker expression. In histograms, each stained sample is shown in orange, and the fluorescence-minus-one (FMO) control is shown in gray.
c, Kaplan-Meier survival curves of tumor-bearing mice treated with CAR T-cells, anti-PD-1 antibody, with perampanel (PER), or with vehicle control (Ctrl) (n = 9 mice per group).
d, Tumor growth curves estimated via BLI. Lines represent individual animal traces, with thick lines indicating mice with tumor clearance to undetectable levels.
P value was calculated using Log-rank test.
e, Gating strategy for flow cytometry to identify CD45.1+ CAR T-cells and CD45.2+ endogenous T-cells within singlet/live/GFP-/CD11b-/CD3+ cell populations (defined as CD3+ T-cells) in brain infiltrating leukocytes (BILs).
f, Scatter plots showing the persistence or absence of CD45.1+ infused CAR T-cells in 5 mice with complete tumor eradication, including 2 from the control (Ctrl) group and 3 from the perampanel (PER) group. Values represent the percentages of CD45.1+ CAR T-cells and CD45.2+ endogenous T-cells within each gate.
g, Scatter plots showing the differentiation status of the detected CD45.1+ CAR T-cells, where two out of three mice in the PER group maintained a memory phenotype, which was not observed in Ctrl group.
h, Bar plots showing the percentages of CD3+ T-cells within CD11b- cells (left), CD45.1+ T-cells within CD3+ T-cells (middle), and CD8+ T-cells within CD45.1+ T-cells (right) (n = 1–2 [Ctrl] and 3 mice [PER]).
i, Bar plots showing the percentages of CD45.1+ CAR T-cells positive for activation or exhaustion markers (n = 1 [Ctrl] and 3 mice [PER]). No statistical testing was performed.
Source data are provided as a Source Data file.

References

1. Krishna, S. *et al.* Glioblastoma remodelling of human neural circuits decreases survival. *Nature* **617**, 599–607 (2023).

2. Hänzelmann, S., Castelo, R. & Guinney, J. GSVA: gene set variation analysis for microarray and RNA-seq data. *BMC Bioinformatics* **14**, 7 (2013).

3. Aibar, S. *et al.* SCENIC: single-cell regulatory network inference and clustering. *Nat. Methods* **14**, 1083–1086 (2017).

4. Noureen, N., Ye, Z., Chen, Y., Wang, X. & Zheng, S. Signature-scoring methods developed for bulk samples are not adequate for cancer single-cell RNA sequencing data. *Elife* **11**, (2022).

5. Ravi, V. M. *et al.* Spatially resolved multi-omics deciphers bidirectional tumor-host interdependence in glioblastoma. *Cancer Cell* **40**, 639-655.e13 (2022).

6. Kueckelhaus, J. *et al.* Inferring histology-associated gene expression gradients in spatial transcriptomic studies. *Nat. Commun.* **15**, 7280 (2024).

7. García-Vicente, L. *et al.* Single-nucleus RNA sequencing provides insights into the GL261-GSC syngeneic mouse model of glioblastoma. *bioRxiv* 2023.10.26.564166 (2023) doi:10.1101/2023.10.26.564166.

8. Aran, D., Hu, Z. & Butte, A. J. xCell: digitally portraying the tissue cellular heterogeneity landscape. *Genome Biol.* **18**, 220 (2017).

9. Genoud, V. *et al.* Responsiveness to anti-PD-1 and anti-CTLA-4 immune checkpoint blockade in SB28 and GL261 mouse glioma models. *Oncoimmunology* **7**, e1501137 (2018).

10. Chuntova, P. *et al.* Novel EGFRvIII-CAR transgenic mice for rigorous preclinical studies in syngeneic mice. *Neuro. Oncol.* **24**, 259–272 (2022).
