# Supplementary material for: Glioma-neuronal circuit remodeling induces regional immunosuppression
Source: Nat Commun. 2025 May 22;16:4770. doi: 10.1038/s41467-025-60074-z (PMC12098748; doi:10.1038/s41467-025-60074-z)
Supplement: Supplementary file 2 — Description of Additional Supplementary Files [file 41467_2025_60074_MOESM2_ESM.pdf]

## Description of Additional Supplementary Files

### Supplementary Data

- **Supplementary Data 1**
  - Results of gene set enrichment analyses (GSEA) of patient scRNA-seq data with MSigDB Hallmark collection comparing HFC vs. LFC within tumor cells (**a**), myeloid cells (**b**), lymphoid cells (**c**), and astrocytes (**d**).

- **Supplementary Data 2**
  - Antibody panels used in multi-color flow cytometry (**a**–**h**).

### Supplementary Movies

- **Supplementary Movie 1**
  - Live-cell calcium imaging from neuron-only condition.

- **Supplementary Movie 2**
  - Live-cell calcium imaging from neurons co-cultured with SB28-TSP1-WT cells.

- **Supplementary Movie 3**
  - Live-cell calcium imaging from neurons co-cultured with SB28-TSP1-KO cells.
