# Supplementary material for: Glioma-neuronal circuit remodeling induces regional immunosuppression
Source: Nat Commun. 2025 May 22;16:4770. doi: 10.1038/s41467-025-60074-z (PMC12098748; doi:10.1038/s41467-025-60074-z)
Supplement: Supplementary file 8 — Reporting Summary [file 41467_2025_60074_MOESM8_ESM.pdf]

nature portfolio

Corresponding author(s): Shawn Hervey-Jumper and Hideho Okada

Last updated by author(s): Apr 2, 2025

Reporting Summary

Nature Portfolio wishes to improve the reproducibility of the work that we publish. This form provides structure for consistency and transparency in reporting. For further information on Nature Portfolio policies, see our Editorial Policies and the Editorial Policy Checklist.

Statistics

For all statistical analyses, confirm that the following items are present in the figure legend, table legend, main text, or Methods section.

n/a	Confirmed	
☐	☒	The exact sample size (*n*) for each experimental group/condition, given as a discrete number and unit of measurement
☐	☒	A statement on whether measurements were taken from distinct samples or whether the same sample was measured repeatedly
☐	☒	The statistical test(s) used AND whether they are one- or two-sided<br>*Only common tests should be described solely by name; describe more complex techniques in the Methods section.*
☐	☒	A description of all covariates tested
☐	☒	A description of any assumptions or corrections, such as tests of normality and adjustment for multiple comparisons
☐	☒	A full description of the statistical parameters including central tendency (e.g. means) or other basic estimates (e.g. regression coefficient) AND variation (e.g. standard deviation) or associated estimates of uncertainty (e.g. confidence intervals)
☐	☒	For null hypothesis testing, the test statistic (e.g. *F*, *t*, *r*) with confidence intervals, effect sizes, degrees of freedom and *P* value noted<br>*Give P values as exact values whenever suitable.*
☒	☐	For Bayesian analysis, information on the choice of priors and Markov chain Monte Carlo settings
☒	☐	For hierarchical and complex designs, identification of the appropriate level for tests and full reporting of outcomes
☐	☒	Estimates of effect sizes (e.g. Cohen's *d*, Pearson's *r*), indicating how they were calculated

Our web collection on statistics for biologists contains articles on many of the points above.

Software and code

Policy information about availability of computer code

Data collection

For mouse spatial transcriptomics data analysis, FASTQ reads were processed using Space Ranger (v1.3.1) and were mapped to the mouse reference (refdata-gex-mm10-2020-A).
For bulk RNA-seq data analysis, FASTQ reads were trimmed using fastp (v0.20.0) and were mapped to the mouse reference genome mm10 (GRCm38.p6) using STAR (v2.7.9a) with transcriptome annotation guidance from gencode.vM25.annotation.gtf. Gene-level expression counts were estimated using stringtie (v2.0). Widefield images were acquired using a Zeiss Axio Imager 2 microscope (20× magnification) with TissueFAXS scanning software (TissueGnostics). Confocal images were acquired using Zeiss LSM780 confocal microscope and Zen imaging software (Carl Zeiss Inc.). For Calcium imaging recording, imaging was performed for 5 min with a time interval of 1 second using a 10× objective and time-lapse images were captured using MetaMorph software. For whole-cell patch clamp recordings, all data were obtained using pClamp 11 acquisition software (Molecular Devices)

Data analysis

For single-cell RNA-seq data analysis, Seurat (v4.0.3) was used for QC, analysis, exploration, and visualization of the data. For mouse transcriptomics analysis, SPATA2 (v0.1.0) was used for analysis, exploration, and visualization of the data. Denoising of the data was performed using the runAutoencoderDenoising() function of the SPATA2 package. For bulk RNA-seq data, differential gene expression analysis and log2 fold change calculation was performed using DESeq2 (v1.32.0). Preranked gene set enrichment analysis (GSEA) was performed using fgsea (1.18.0). Widefield and confocal image analyses was done using Fiji ImageJ (v2.9.0). Mouse survival analysis was performed using the log-rank test from the R package survival, and Kaplan-Meier survival curves were visualized using the R package survminer. Statistical analysis and data visualization were performed with R (v4.1.2). For Calcium imaging recording, data was analyzed using FluroroSNNAP software. For whole-cell patch clamp recordings, data were analyzed using Easy Electrophysiology software.

Regarding covariate control, since single-cell RNA sequencing was performed on dissociated tumor-derived cells, covariates such as age and

sex were not explicitly controlled. However, major sources of technical variability, including batch effects, were assessed and corrected using methods, such as Harmony.

Code availabilty:
The codes used for data analysis can be found at Github: https://github.com/t-nejo/Glioma-neuron-immune-crosstalk.

For manuscripts utilizing custom algorithms or software that are central to the research but not yet described in published literature, software must be made available to editors and reviewers. We strongly encourage code deposition in a community repository (e.g. GitHub). See the Nature Portfolio guidelines for submitting code & software for further information.

Data

Policy information about availability of data

All manuscripts must include a data availability statement. This statement should provide the following information, where applicable:

- Accession codes, unique identifiers, or web links for publicly available datasets
- A description of any restrictions on data availability
- For clinical datasets or third party data, please ensure that the statement adheres to our policy

The 10x Visium mouse spatial transcriptomics data and mouse in vivo tumor model RNA-seq data newly generated in this study are available through the NCBI Gene Expression Omnibus (GEO) website under accession numbers GSE289934 [https://www.ncbi.xyz/geo/query/acc.cgi?acc=GSE289934] and GSE289935 [https://www.ncbi.xyz/geo/query/acc.cgi?acc=GSE289935], respectively. The publicly available data used in this study are available as follows: human glioblastoma single-cell RNA-seq: in the GEO database under accession code GSE223065 [https://www.ncbi.xyz/geo/query/acc.cgi?acc=GSE223065]14; mouse glioma SB28 RNA-seq: in the GEO database under accession code GSE127075 [https://www.ncbi.xyz/geo/query/acc.cgi?acc=GSE127075]38; mouse glioma GL261 RNA-seq: in the GEO database under accession code GSE94239 [https://www.ncbi.xyz/geo/query/acc.cgi?acc=GSE94239]; normal mouse brain bulk RNA-seq: in the EMBL-EBI ArrayExpress database under accession code E-MTAB-6081 [https://www.ebi.ac.uk/arrayexpress/files/E-MTAB-6081]79; 10x Visium mouse brain spatial transcriptomics data of GL261: in the GEO database under accession code GSE245263 [https://www.ncbi.xyz/geo/query/acc.cgi?acc=GSE245263]40. Human glioblastoma 10x Visium spatial transcriptomics data are available using the R package SPATAData [https://github.com/theMILOlab/SPATAData]34,35, as described earlier. All other study data are included in the manuscript and/or supporting information. Source data are provided with this paper.

Research involving human participants, their data, or biological material

Policy information about studies with human participants or human data. See also policy information about sex, gender (identity/presentation), and sexual orientation and race, ethnicity and racism.

Reporting on sex and gender	The human subject data used in this work are all from adult males/females diagnosed with high-grade glioblastoma. Our findings apply to both sexes. A detailed information of patient's sex and age used in this study has been reported previously (Krishna S. et al., Nature 617, 599–607 (2023)).
Reporting on race, ethnicity, or other socially relevant groupings	N/A
Population characteristics	The human subject data used in this work were all from adult makes/females diagnosed with high-grade glioblastoma. Clinical information of the patients has been reported previously (Krishna S. et al., Nature 617, 599–607 (2023)).
Recruitment	All study participants were individuals seeking care for presumed diffuse glioma at University of California, San Francisco. Each participant in this study was recruited from a prospective registry of adults aged 18–85 with newly diagnosed frontal, temporal, and parietal high-grade glioma. Inclusion criteria was patients with suspected brain tumor on magnetic resonance imaging (MRI). Patients were recruited by a brain tumor center clinical research coordinator who was not involved in clinical patient care in order to limit the potential for enrollment bias. More detailed information of the patient recruitment has been reported previously (Krishna S. et al., Nature 617, 599–607 (2023)).
Ethics oversight	This study complied with all relevant ethical regulations and was approved by the University of California, San Francisco (UCSF) institutional review board for human research (UCSF CHR 17-23215).

Note that full information on the approval of the study protocol must also be provided in the manuscript.

Field-specific reporting

Please select the one below that is the best fit for your research. If you are not sure, read the appropriate sections before making your selection.

☒ Life sciences ☐ Behavioural & social sciences ☐ Ecological, evolutionary & environmental sciences

For a reference copy of the document with all sections, see nature.com/documents/nr-reporting-summary-flat.pdf

Life sciences study design

All studies must disclose on these points even when the disclosure is negative.

Sample size	Patients presented for resection of glioblastoma who gave consent for tumor sampling for research were included in the study, which was approved by the institutional review board (17-23215). No sample size calculation was performed, and all samples from patients meeting

	inclusion and exclusion criteria were included. Sample selection criteria were detailed in the Methods section as well as reported previously (Krishna S. et al., Nature 617, 599–607 (2023)).
Data exclusions	No data were excluded from the analyses.
Replication	All experiments were performed at least in triplicates and measurements were reproducible with biological replicates performed on separate cohort of animals and cells.
Randomization	All animals intracranially inoculated with each cell line used for survival experiments and immunological assessments were analyzed in the same way – no randomization was necessary. For pharmacological study, mice inoculated with SB28-TSP1-WT cells were not randomized but equally assigned to each treatment group based on the body weight distributions, and orally treated with perampanel (PER) or corresponding vehicle. For immunotherapy study, based on BLI-estimated tumor size on day 7, mice were randomized into either CAR T + ICB + PER group or CAR T + ICB + vehicle control (Ctrl) group.
Blinding	In mouse survival experiments testing drug treatment (PER) or immunotherapy (CAR T + ICB + PER), the judgment of euthanasia was indicated by the veterinarian staff members who were blinded to the study groups.

Reporting for specific materials, systems and methods

We require information from authors about some types of materials, experimental systems and methods used in many studies. Here, indicate whether each material, system or method listed is relevant to your study. If you are not sure if a list item applies to your research, read the appropriate section before selecting a response.

Materials & experimental systems

n/a	Involved in the study	
☐	☒	Antibodies
☐	☒	Eukaryotic cell lines
☒	☐	Palaeontology and archaeology
☐	☒	Animals and other organisms
☒	☐	Clinical data
☒	☐	Dual use research of concern
☒	☐	Plants

Methods

n/a	Involved in the study	
☒	☐	ChIP-seq
☐	☒	Flow cytometry
☒	☐	MRI-based neuroimaging

Antibodies

Antibodies used

Primary antibodies used in western blotting:
anti-Thrombospondin-1 clone A6.1 (1:500; Thermo Fisher Scientific; MA5-13398; clone A6.1)
anti-GAPDH clone 14C10 (1:1000; Cell Signaling Technologies, #2118; clone 14C10)

Secondary antibodies used in western blotting:
anti-mouse IgG and anti-rabbit IgG HRP-linked antibodies (1:5000; Cell Signaling Technologies; #7076 and #7074, respectively).

Primary antibodies used in immunofluorescence:
1% TruStain FcX PLUS anti-mouse CD16/32 Antibody (1:100; BioLegend; 156604)
anti-MAP2-Rabbit pAb (1:500, Synaptic Systems; 188 003)
anti-Synapsin-1 Mouse mAb (1:200, Synaptic Systems; 106 011; clone 46.1)
anti-Homer1 Guinea Pig pAb (1:200, Synaptic Systems; 160 004)
anti-Thrombospondin-1 Rabbit pAb (1:200, ab85762; Abcam)

Secondary antibodies used in immunofluorescence:
Goat Anti-Rabbit IgG AF594 (Abcam, ab150084)
Goat anti-Mouse IgG AF514 (Invitrogen, A-31555)
Goat anti-Guinea Pig IgG (H+L) AF647 (1:250; Invitrogen; A-21450),
Goat anti-Rabbit IgG H&L AF647 (1:250; Abcam; ab150083).

Antibodies used in flow cytometry are provided in Supplementary Table S2.

Validation

All the antibodies used in the study were purchased from commercial vendors and were validated by the manufacturers, and used in other studies.

Primary antibodies used in western blotting:
anti-Thrombospondin-1 clone A6.1 (1:500; Thermo Fisher Scientific; MA5-13398; clone A6.1)
Cambier S, Gline S, Mu D, et al. Integrin alpha(v)beta8-mediated activation of transforming growth factor-beta by perivascular astrocytes: an angiogenic control switch. Am J Pathol. 2005;166(6):1883-1894. doi:10.1016/s0002-9440(10)62497-2. Validated in FACS, ICC/IF, IHC (P), IM, IP, and WB by provider.

anti-MAP2 Rabbit mAb (1:500, Synaptic Systems; 188 003)
Amer-Sarsour F, Falik D, Berdichevsky Y, Kordonsky A, Eid S, Rabinski T, Ishtayeh H, Cohen-Adiv S, Braverman I, Blumen SC, Laviv T,

Prag G, Vatine GD, Ashkenazi A. Disease-associated polyalanine expansion mutations impair UBA6-dependent ubiquitination. EMBO J. 2024 Jan;43(2):250-276. doi: 10.1038/s44318-023-00018-9. ICC; tested species: mouse.

anti-Synapsin-1 Mouse mAb (1:200, Synaptic Systems; 106 011)
Kuijpers M, Kochlamazashvili G, Stumpf A, et al. Neuronal Autophagy Regulates Presynaptic Neurotransmission by Controlling the Axonal Endoplasmic Reticulum [published correction appears in Neuron. 2022 Feb 16;110(4):734]. Neuron. 2021;109(2):299-313.e9. doi:10.1016/j.neuron.2020.10.005. Validated in ICC, IHC, IHC-P, IP, and WB by provider. K.O. validated.

anti-Homer1 Guinea Pig pAb (1:200, Synaptic Systems; 160 004)
Meijer M, Rehbach K, Brunner JW, et al. A Single-Cell Model for Synaptic Transmission and Plasticity in Human iPSC-Derived Neurons. Cell Rep. 2019;27(7):2199-2211.e6. doi:10.1016/j.celrep.2019.04.058. Validated in ICC, IHC, IHC-P, IP, and WB by provider.

anti-Thrombospondin-1 Rabbit pAb (1:200, ab85762; Abcam)
Diéguez-Hurtado R, Kato K, Giaimo BD, et al. Loss of the transcription factor RBPJ induces disease-promoting properties in brain pericytes. Nat Commun. 2019;10(1):2817. Published 2019 Jun 27. doi:10.1038/s41467-019-10643-w. Validated in IHC-P, ICC/IF, and WB by provider.

Validation information for the antibodies used in flow cytometry are provided in Supplementary Table S2.

Eukaryotic cell lines

Policy information about cell lines and Sex and Gender in Research

Cell line source(s): The eukaryotic cell line SB28 was generated in the Hideho Okada lab. The cell line is deposited at the German Collection of Microorganisms and Cell Cultures (DSMZ) repository as SB28-Ohlfest (ACC 880) and made publicly available.

Authentication: Species-level identification was carried out by mitochondrial Cytochrome C Oxidase Subunit 1 (COI) DNA barcoding according to standard ANSI/ATCC ASN-0003-2015 and revealed Mus musculus species.

Mycoplasma contamination: All cell cultures are routinely tested for mycoplasma contamination and all cultures were tested negative.

Commonly misidentified lines (See ICLAC register): No commonly misidentified lines were used.

Animals and other research organisms

Policy information about studies involving animals; ARRIVE guidelines recommended for reporting animal research, and Sex and Gender in Research

Laboratory animals: C57BL/6J mice (Jackson Laboratory, 000664) female mice were used between 5–7 weeks of age at the time of intracranial tumor inoculation. Mouse housing condition have been described in the manuscript.

Wild animals: N/A

Reporting on sex: In this study, only female mice were used between 5–7 weeks of age at the time of intracranial tumor inoculation. Male mice were not included, as sex-based differences were not specifically investigated. For experiments using neonatal mouse cortical neurons, sex was not considered.

Field-collected samples: N/A

Ethics oversight: All the mouse studies were performed following the protocol approved by the Institutional Animal Care and Use Committee (IACUC) of UCSF.

Note that full information on the approval of the study protocol must also be provided in the manuscript.

Flow Cytometry

Plots

Confirm that:

☒ The axis labels state the marker and fluorochrome used (e.g. CD4-FITC).

☒ The axis scales are clearly visible. Include numbers along axes only for bottom left plot of group (a 'group' is an analysis of identical markers).

☒ All plots are contour plots with outliers or pseudocolor plots.

☒ A numerical value for number of cells or percentage (with statistics) is provided.

Methodology

Sample preparation: Brain-infiltrating leukocytes (BILs) were isolated by density-gradient centrifugation using Percoll (GE Healthcare Life Sciences, 17089101), as previously described53. After isolation, single-cell suspensions of BILs (0.5–1×106 cells) were stained with

Zombie Aqua™ dye (BioLegend, 423102) to discriminate between live and dead cells. Fc receptor blocking was performed using TruStain FcX PLUS anti-mouse CD16/32 Antibody (BioLegend, 156604) prior to staining with fluorophore-conjugated antibodies at the concentrations recommended by the manufacturers. Fluorescence minus one (FMO) controls were used to determine accurate gating. The antibody panels are listed in Supplementary Tables S2a–h. Data were acquired using an Attune NxT flow cytometer (Thermo Fisher Scientific) or Cytek Aurora Spectral Flow Cytometry (Cytek Biosciences) and analyzed using FlowJo software (Tree Star, version 10.8.1).

Instrument	Data were acquired using an Attune NxT flow cytometer (Thermo Fisher Scientific) or Cytek Aurora Spectral Flow Cytometry (Cytek Biosciences).
Software	Data was analyzed using FlowJo software (Tree Star, version 10.8.1).
Cell population abundance	The description was provided in the extended data.
Gating strategy	Fluorescence minus one (FMO) controls were used to determine the accurate gating. Using the FSC/SSC gating, debris was removed, and the single alive cells were gated. Each population was gated based on the surface or intracellular markers as described in the manuscript. Details are provided in the main text and the extended data.

☒ Tick this box to confirm that a figure exemplifying the gating strategy is provided in the Supplementary Information.
